# Predictive validity of the UKCAT for medical school undergraduate performance: a national prospective cohort study

**DOI:** 10.1186/s12916-016-0682-7

**Published:** 2016-09-26

**Authors:** Paul A. Tiffin, Lazaro M. Mwandigha, Lewis W. Paton, H. Hesselgreaves, John C. McLachlan, Gabrielle M. Finn, Adetayo S. Kasim

**Affiliations:** 1 Department of Health Sciences, University of York, Heslington, York, YO10 5DD UK; 2 Institute of Health and Wellbeing, University of Glasgow, 1 Lilybank Gardens, Glasgow, G12 8RZ UK; 3 Medical Education, School for Medicine, Pharmacy and Health, Durham University Queen’s Campus, Stockton-on-Tees, TS17 6BH UK; 4 Hull York Medical School, Heslington, York, YO10 5DD UK; 5 Wolfson Research Institute for Health and Wellbeing, Durham University Queen’s Campus, University Boulevard, Stockton-on-Tees, TS17 6BH UK

**Keywords:** Predictive validity, Medical selection, Undergraduate performance, Aptitude testing

## Abstract

**Background:**

The UK Clinical Aptitude Test (UKCAT) has been shown to have a modest but statistically significant ability to predict aspects of academic performance throughout medical school. Previously, this ability has been shown to be incremental to conventional measures of educational performance for the first year of medical school. This study evaluates whether this predictive ability extends throughout the whole of undergraduate medical study and explores the potential impact of using the test as a selection screening tool.

**Methods:**

This was an observational prospective study, linking UKCAT scores, prior educational attainment and sociodemographic variables with subsequent academic outcomes during the 5 years of UK medical undergraduate training. The participants were 6812 entrants to UK medical schools in 2007–8 using the UKCAT. The main outcome was academic performance at each year of medical school. A receiver operating characteristic (ROC) curve analysis was also conducted, treating the UKCAT as a screening test for a negative academic outcome (failing at least 1 year at first attempt).

**Results:**

All four of the UKCAT scale scores significantly predicted performance in theory- and skills-based exams. After adjustment for prior educational achievement, the UKCAT scale scores remained significantly predictive for most years. Findings from the ROC analysis suggested that, if used as a sole screening test, with the mean applicant UKCAT score as the cut-off, the test could be used to reject candidates at high risk of failing at least 1 year at first attempt. However, the ‘number needed to reject’ value would be high (at 1.18), with roughly one candidate who would have been likely to pass all years at first sitting being rejected for every higher risk candidate potentially declined entry on this basis.

**Conclusions:**

The UKCAT scores demonstrate a statistically significant but modest degree of incremental predictive validity throughout undergraduate training. Whilst the UKCAT could be considered a fairly crude screening tool for future academic performance, it may offer added value when used in conjunction with other selection measures. Future work should focus on the optimum role of such tests within the selection process and the prediction of post-graduate performance.

**Electronic supplementary material:**

The online version of this article (doi:10.1186/s12916-016-0682-7) contains supplementary material, which is available to authorized users.

## Background

For many years, access to medical school worldwide has been determined primarily on educational attainment, measured by secondary (high) school grades. This has been an accepted approach as it generally reliably predicts later academic performance in medical school and later postgraduate clinical education [1]. In the UK, as elsewhere, there is a high competition ratio for places at medical school, with around 11 high achieving applicants for each place [2]. Internationally, this competition has encouraged the development of aptitude tests as part of the selection of future doctors. Specifically, aptitude tests seek to quantify the cognitive (and increasingly personal) attributes that are considered desirable in a future doctor and important to successfully completing both undergraduate and postgraduate training as well as practising effectively.

In England, secondary school academic achievement and entry to university is usually focussed on General Certificate of Education Advanced-level grades (‘A-levels’). There has been a fairly continual increase in the average grades awarded at A-level over the last two decades. This ‘grade inflation’ means that prior (or predicted) educational attainment measures are losing their discriminatory power to identify future ability to perform in medical school [3]. Thus, candidates are restricted in the scores attainable from secondary school qualifications such that they cannot demonstrate capability beyond other applicants achieving the same grades. UK universities tend to accept only the first top three grades achieved at A-levels, reducing the variation in accepted entry qualifications further. These top grades are converted from alphabetic form (e.g. A–E) grades to a ‘tariff’ points score by the Universities and Colleges Admissions Service (UCAS) for the UK. Each grade is worth a set amount of tariff points, irrespective of the subject. However, for standard entry (as opposed to graduate entry or extended courses with a ‘pre-medicine’ year of study) medical schools usually require at least two of the sciences to have been studied at advanced level and also a certain level of achievement at mathematics. Thus, at the time of the study, the maximum achievable UCAS tariff score for ‘best of three’ A-levels was 360 points (equivalent to three A grades, each worth 120 points). In Scotland and Ireland the situation differs slightly. In Scotland, school students wishing to apply for the more competitive university courses typically sit ‘Scottish Higher’ exams in five subjects in their fifth year, followed by an additional two or three at ‘Advanced Higher’ level in their sixth year (the latter being more comparable with A-levels). At the time of this study, most Scottish medical schools would have required mostly ‘A’ grades at Higher level. UK medical schools outside Scotland generally also required Scottish Certificate of Education candidates to study up to three subjects at Advanced Higher or Higher level, with either AAA or AAB grades, and with subject specification (generally Chemistry and Biology), but this varied amongst medical schools. In Ireland, students intending to study medicine would tend to take seven Irish Leaving Certificate Examinations (ILEs), with an emphasis on science subjects. Medical school entry requirements, at the time, for applicants with Irish advanced educational qualifications, would have been ‘A’ grades for the best of six of the subjects taken; for ILEs the A grade is split into two levels, A1 and A2, with A1 being the highest achievable grade worth 90 UCAS tariff points. Usually, this needed to include at least two science subjects. Most Irish medical schools also required a specified standard in English, mathematics, Irish, and a third language.

In practice, many applicants to medical school are predicted (or have obtained) top ‘A’ grades at their A-level exams (i.e. AAA, or A*A*A* following introduction of the ‘A star’ grade in 2010) or the Scottish or Irish equivalents. Consequently, one rationale for the introduction of aptitude tests in selection is to provide a continuous and uncensored metric of ability to inform selection decisions (i.e. one without upper range restriction), thus providing a way of differentiating applicants at the top range of academic ability.

The second main rationale for the introduction of the UKCAT into medical selection in 2006 was as a component of attempts to widen access to medical careers to those from under-represented and less advantaged backgrounds. Indeed, in the UK, medicine has been singled out as a profession where very little progress has been made in recent decades with regards to social mobility [4]. Traditional metrics of educational achievement (including predicted A-level grades) and personal statements may favour those attending fee paying or otherwise selective schools [5, 6]. Previous research suggests that the use of the UKCAT score as a threshold for interview or place offer may mitigate against the disadvantage encountered by certain underrepresented groups when applying to medical school [7]. Moreover, the UKCAT may be somewhat less sensitive to school-type attended when compared to A-levels [8]. The UKCAT is also intended to capture constructs that may not be well evaluated using traditional academic measures (such as decision-making ability).

The UKCAT is currently used by 26 of the 34 UK medical schools and consists of four scales designed to evaluate different aspects of cognitive ability using a selected response format, namely verbal reasoning, quantitative reasoning, abstract reasoning and decision making (formerly decision analysis). For the ‘verbal reasoning’ sub-test, candidates are presented with a series of passages of text and are asked to draw inferences from them. For ‘quantitative reasoning’, candidates have to solve numerical problems, using an on-screen calculator, if required. These problems are framed in a practical, applied way (for example, calculating the total cost of renting a boat for a specified circumstance). In ‘abstract reasoning’, a series of items involving shapes are presented and the candidate must select the correct response by recognising sequences and patterns. For ‘decision analysis’, candidates were presented with a scenario and a set of codes (e.g. A = ‘Always’, B = ‘Good’, C = ‘Lawyer’, etc.). The test items consisted of statements (e.g. ‘Good lawyers are always loyal to their firm’). The correct set of codes that best reflected the meaning of the statement had to be selected. In 2016, the ‘decision analysis’ sub-test was replaced by a different ‘decision making’ scale. For the other sub-tests, sample items are publically available at the UKCAT website [9]. Previous analysis suggests a moderate to high degree of correlation exists between the scores on the UKCAT cognitive subtests; on factor analysis, correlations between the postulated factors underlying the four scale scores ranged from 0.32 (‘verbal reasoning’ and ‘abstract reasoning’) to 0.75 (‘decision analysis’ and ‘quantitative reasoning’) [10]. More recently, in addition to the cognitive scales, Situational Judgement Testing has been introduced, although this is not included in the present study.

Since its conception, the UKCAT has attracted criticism, not least because candidates must (at the time of writing) pay a minimum fee of £65 to sit the test, although bursaries are available for economically disadvantaged testees. Thus, in order to justify the place of the test within medical selection it is important to establish the extent to which an aptitude test adds value in the process, particularly above and beyond that provided by conventional educational metrics, such as predicted or obtained A-level grades [11].

Ultimately, an aptitude test should be able to accurately predict occupational performance. However, given the length of medical training and the availability of data on clinical performance and outcomes, inevitably early evidence of the validity for selection methods are sought via performance in undergraduate settings. Moreover, there is evidence of an association between medical school academic performance and later career success. For example, one American study highlighted evidenced of a link between later professional misconduct and unprofessional behaviour and poor academic performance during medical training [12]. Thus, such tests may have some potential to predict later difficulties in practice, albeit indirectly via the estimation of cognitive ability.

Findings from a previous national study reported a modest but statistically significant ability of the UKCAT scores to predict undergraduate performance in year 1 (the standardised regression coefficients were up to 0.18 in magnitude). This effect persisted to some extent even after correction for educational attainment (best of three A-levels or equivalent); the adjusted regression coefficient for the prediction of overall performance in year 1 from total UKCAT score was cited as approximately 0.101, with an associated *P* value of less than 0.001 [13]. Other, smaller scale, regionally based studies suggest the ability of UKCAT scores to predict undergraduate academic achievement may persist into the clinical years of undergraduate medical training [14–16]. However, these latter, locally based studies have not attempted to correct for the effects of school educational achievement when evaluating the predictive validity of the UKCAT.

In North America, longitudinal analysis of the Medical Colleges Aptitude Test (MCAT) have reported a decline in the ability of the test to predict academic undergraduate performance between the pre-clinical and clinical years [17, 18]. However, McManus highlights that the MCAT is largely a test of substantive understanding of science, as well as attainment (but not aptitude) [19]. This observation may explain the reduction in MCAT’s predictive power in clinical years of education, when achievement may be increasingly based on attributes such as reasoning, decision making and conscientiousness.

In order to further explore the predictive validity of the UKCAT, we present evidence from the first national study to include longitudinal outcomes throughout all 5 years of a UK medical degree. By examining outcomes at different time points within the cohorts we can determine how the UKCAT performs as a predictor at each stage in medical undergraduate assessment of both theory (i.e. semantic knowledge-based tests) and skills (assessments that are presumed to have a procedural component to them). In addition to this, we will account for the possible effects of traditional measures of academic attainment (e.g. A-level performance) and socio-demographic background factors (e.g. school type attended) as variables that correlate both with UKCAT scores and undergraduate performance, thereby assessing UKCAT's explanatory value over and above that provided by these known confounding factors. However, in this report we focus on the adjustment for prior educational attainment. This is because this factor is the only one, at the time of the study, considered by current selection processes that could also be quantified in a relatively standardised way nationally (in contrast to personal statements and interview ratings, etc.).

The UKCAT scores (most usually the summed total scores) are commonly used by medical schools as a threshold to offer a candidate an interview or a place [20]. In order to explore the potential impact of this approach to test score use, we take a novel, ‘evidence-based selection’ approach to the analysis, introducing the new concept of ‘number needed to reject’ (NNR). This approach involves simulating outcomes for candidates who did not have university progression data available (e.g. they failed to obtain a place at medical school) and treats the UKCAT as a screening tool. Thus, our findings will have implications for the way the test is used within the overall medical selection process.

## Methods

Data were available for candidates who completed the UKCAT and entered a UKCAT consortium medical school (i.e. one using the test in selection) between 2007 and 2013. However, only data for 6812 students in two cohorts who entered medical school in 2007 and 2008 were used in the analysis. This is because, compared to other cohorts, these two earlier cohorts had relatively high levels of data availability throughout the 5 year undergraduate period of study (Table 1), thus reducing the risk of attrition bias being introduced. We excluded applications specifically relating to graduate entry or “widening participation” courses (i.e. those where applicants held a degree and lower than usual A-level or equivalent grades would be accepted, respectively) as they were considered a potential source of confounding. It is important to note that the number of universities contributing to the dataset at each year varied somewhat (Table 1).

**Table 1:** Study attrition rates (mainly due to missing data) in relation to the original 2007 and 2008 entry cohorts

	Year of entry = 2007			Year of entry = 2008		
Academic Year	Number of universities	Number of students	Percentage of attrition	Number of universities	Number of students	Percentage of attrition
1	16	2821	–	18	3604	–
2	17	2731	3.19	18	3522	2.28
3	16	2509	11.06	16	3024	16.09
4	14	2080	26.27	13	2540	29.52
5	11	1614	42.79	7	1280	64.48

### Data preparation

**Fig. 1:**
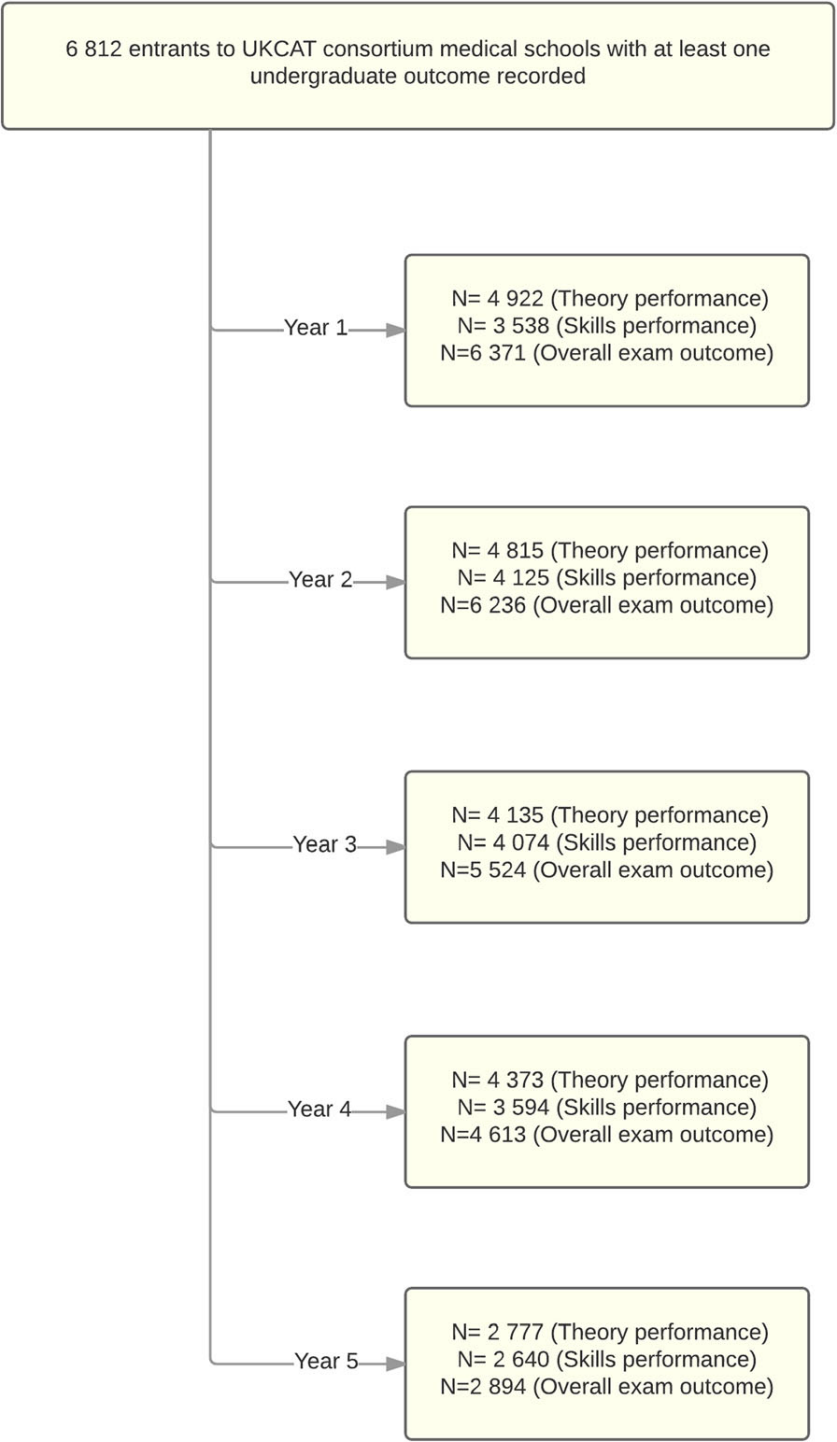
Chart showing the flow of data through the study

In the UK, applications to university are managed via the UCAS. The data were linked between the UCAS and UKCAT databases at the individual level using the unique UCAS ID for university applicants by the UKCAT data managers. The UKCAT ID and other potential identifiers (such as name and date of birth) were also potentially available to facilitate and confirm correct linkage had occurred, if required. The data were then released to the research team in de-identified form. Figure 1 depicts the data included in the final analyses.

#### Outcome variables

A standard undergraduate medical course in the UK is of 5 years duration. Progression through successive years is usually conditional on satisfactory academic performance, as indexed by in-course assessment and regular summative exams. When the UKCAT was introduced, an agreement with the universities that make up the consortium using the test was made to provide admission and progression data in relation to candidates who had taken the test and entered the individual medical and dental schools, though several schools did not participate in this data-sharing agreement. In addition, some participating universities were only able to provide partial datasets due to resource constraints.

For the purposes of the present study, the main dependent variables used were the scores at skills- and theory-based end of year assessments. Medical schools chose how to define student scores for theory or skills, referring to their undergraduate curricula. More detailed descriptions of these definitions were not available due to the variability in course and assessment content and the limited resources available to UKCAT at the time for the data collection exercise. Thus some variability across medical school in terms of the nature of these two categories of assessment might be expected. The relevant assessment scores for students were collected by the UKCAT office using a standard spreadsheet for each academic year. These progression data were then uploaded to the UKCAT database using a web-based interface provided by the UKCAT data managers, based at the Health Informatics Centre at the University of Dundee. The assessment scores were originally provided in percentage forms (of maximum marks achievable) and converted to standardised z-scores within each institution and year group. This standardisation was carried out in order to increase the comparability of scores across institutions and cohorts, thus minimising the impact of any variability in the nature of assessments that may have occurred. The overall academic outcome for each course year was also reported by medical schools. Thus, whether a student passed their end of year exams, in contrast to another academic outcome (e.g. needing to resit) was analysed as a separate, dichotomous outcome variable.

#### Predictor variables

Applicants wishing to enter a UKCAT consortium medical school must sit the test the preceding year (e.g. from July 1 to October 5, 2016, for entry in October 2017). There is no limit on the number of times the test may be taken, though it can only be sat once for each admission cycle. For the present study the UKCAT scores and year of sitting were available. The four scale scores and total scores were standardised as z-scores within each cohort of test-takers (including unsuccessful applicants) for that year. For those that had taken the UKCAT multiple times, the scores from the most recent sitting were used. This is because these will have been the level of UKCAT performance used by the admitting universities to make the decision to offer a place.

As with our previous studies of the UKCAT, we created a continuous metric of academic performance that included Irish and Scottish qualifications as well as A-levels [7]. This was done by summarising the examination results as a percentage of the maximum achievable UCAS tariff scores that could be obtained. Standardised z scores were then derived within students for each nationality (i.e. we compared all those taking Scottish “higher” qualifications against each other). We included only the first three highest grades, excluding General Studies. In practice, the vast majority of subjects taken at A-level were science or mathematics (in keeping with the entry requirements for medicine). In the case of Scottish Highers and Irish leaving certificates, we used the best of five or six exams, respectively. As with the A-levels, Irish and Scottish exams were mostly taken in mathematics and the sciences, though significant numbers of students also took other subjects such as English, French and geography. Irish was also frequently studied for the ILE (this is a common entry requirement to Irish medical schools). Only the grades at first sitting were retained, in all cases. We also excluded data from candidates who did not have the minimum number of advanced qualifications required (for example, fewer than three A-level passes). Thus, our management of the advanced educational qualification data emulated the approach typically used by UK medical schools in appraising predicted or achieved secondary school qualifications. The UKCAT database records reported socioeconomic status using a simplified version of the socioeconomic classification system used by the National Office for Statistics [21]. As with our previous research in this area, we classified those who gave themselves a socioeconomic classification rating of four or more as being from a ‘non-professional’ background, ethnicity was dichotomised into White and Non-white, and schools into selective (independent and grammar schools) and non-selective (state schools and sixth form colleges). Age was dichotomised into those who were 21 or older at medical school entry (i.e. ‘mature students’) and those that were younger on admission, using the date of birth. Applicants who may have special educational needs (SENs) may also apply for SEN status for the purposes of sitting the UKCAT. This permits such candidates additional time to complete the test and the SEN status of applicants was included in the study dataset.

### Data analysis

A linear mixed modelling approach was used to analyse the data in relation to the continuous outcomes (skills and theory scores). This allowed for the effects of predictors to vary across the individual medical schools in which students were nested. Univariable analysis was conducted in order to guide variable selection for the multivariable models; the sociodemographic variables that had a regression coefficient with an associated *P* value ≤ 0.1 were included. Once the final variables were selected, potential interactions between predictors were evaluated. Model building used a backward stepwise approach, using likelihood ratio tests, whereby non-significant (*P* > 0.05) covariates were eliminated. Separate multivariable models were developed for each UKCAT scale (including the total score) and each exam type (skills or theory) for each subsequent year of medical school (50 models in total), controlling for the potential effects of relevant educational and sociodemographic factors. In addition, an additional 50 separate models were run which only controlled for the effect of advanced qualification on the relationship between the UKCAT scores and the outcome, in order to evaluate the incremental predictive validity of the UKCAT above that provided by conventional educational attainment.

In order to model the prediction of the UKCAT scores for the odds of passing a medical school year at first attempt (versus resitting the exam or year) a Generalised Estimating Equation (GEE) framework with a logit link function was used. This was analogous to a three-level multilevel logistic regression model (with clustering at university and student level), but in this case GEE was used as it produces population-averaged regression coefficients. In contrast, a traditional multilevel model, using maximum likelihood estimation would produce regression coefficients which would be cluster-specific. As the chances of failing a year decrease with time, a temporal variable was included in the model. Five separate models were developed, relating to the four scales and total score of the UKCAT.

#### Missing data handling

Missing values inevitably pose a challenge when analysing observational data. The risk of bias is related to the underlying mechanism for the missingness [22]. Traditionally missing data can be classified as: ‘Missing Completely at Random’, where the pattern of missingness is arbitrary and purely due to chance; ‘Missing at Random’ (MAR) where the absent values are related to the variables that can be observed; and ‘Missing not at Random’ (MNAR), where the missing values are neither due to the workings of pure chance nor associated with the values of the observed data [23]. Although there are a number of approaches to dealing with missing data in statistical analyses, the use of multiple imputation is one of the most commonly employed [24]. In imputation, plausible values for missing data are drawn, at random, from a distribution (often normal (Gaussian) in shape) of possible ones. The shape of the distribution (in the case of a normal distribution specified by the mean and variance) from which the values are drawn can be informed by the observed data, under the assumption that the data are MAR (i.e. the unobserved values are related to those observed). In the case of multiple imputation, a number of data sets are created where the missing values have been imputed. These data sets are analysed separately and then the results are combined. By imputing values for missing data more observations can be included in analysis, and hence a potentially greater study power can be achieved. For example, ordinarily, in a multiple regression, if a student had a missing value for age, and this was a covariate in the analysis, the remaining data from that individual could not be included in the modelling. In addition, by comparing the results from the analysis of data sets with and without imputed values we are able to infer, to some extent, whether the missingness is likely to be MAR or MNAR. In this regard, we analysed the datasets with missing values using Full Information Maximum Likelihood, which assumes the unobserved values are MAR. We then analysed the multiply imputed datasets (which also assumes missing values are MAR) and compared the two sets of results. Though not conclusive, where the results from imputed datasets differ little from those with no imputed missing values, then the data are more likely to be MAR than MNAR, thus we can be more confident in the validity of our findings, and vice versa [24].

Thus, for the purposes of this study, in order to assess and minimise the impact of missing data on the main results, multiple imputation was used as a sensitivity analysis. In this sense, the approach was used in order to explore the potential impact of the missing values on our main findings, and hence the level of certainty regarding any inferences drawn from them. According to our data exploration the missingness pattern was non-monotone (Additional file 1: Supplementary and Technical Appendix; Tables S1 to S4). This means that missing data could occur, for example, for a student in year 2, but then be non-missing in subsequent years. Therefore Multiple Imputation through Chained Equations, also known as Full Conditional Specification, was utilised. This approach fills in missing values in multiple variables iteratively by using chained equations to sequentially impute absent values, beginning with the most complete variables first [25]. A series of sensitivity analyses were conducted, comparing the results from 10, 20 and 30 imputed versus non-imputed datasets. The sensitivity analyses findings, comparing the results from imputed and non-imputed datasets, are contained in Additional file 1: Technical Appendix (see Additional file 1: Figures S1 and S2; see also Additional file 1: Tables S5 to S9), along with a discussion of the implications for our study.

#### Conceptualising the UKCAT as a screening tool: Receiver Operator Characteristic (ROC) curve analysis

In order to take an ‘evidence-based selection’ approach, we treated the UKCAT total score as a screening test [26], with failure to pass at least 1 year of medical school at first attempt as the outcome of interest.

We could not observe performance in many UKCAT candidates. In many cases, this was due to the candidate failing to secure a place at medical school, though some will have gone to non-UKCAT medical schools (no data were available to clarify the reason). In order to address this issue, and obtain some evidence of the likely impact of using the UKCAT as part of the selection process, we used a two stage imputation process. Firstly, we used a single imputation for any missing progression outcomes in the entrants. The imputation value was based on a logistic regression with advanced qualifications, ethnicity, gender and preceding exam outcomes as the predictors. From this, we calculated an estimate of which entrants had (or were likely to have) passed all their years at medical school at first sitting. Secondly, a single binary summary of progression outcomes (passed all years at first sitting or not) was imputed for those who sat the UKCAT in 2006 and 2007, but where there was no record of them having entered a UKCAT consortium university. This allowed us to generate a ROC curve for the UKCAT as a screening instrument for adverse academic outcomes in medical school and, thus, at least crudely estimate the potential effectiveness of the UKCAT in this respect. Consequently, we were able to inspect the ROC curve for the UKCAT total score to determine if an optimum threshold existed to accurately identify students who had failed (or were predicted to fail) to pass at least 1 year at first attempt. In addition, we were also able to explore the potential impact of selecting relatively low or high cut-points on the UKCAT total score.

The data analyses were conducted using SAS version 9.4 [27]. The only exception to this was that the ROC curve analysis and related imputations were carried out using Stata MP version 14.1 [28].

## Results

Figure 1 depicts the flow of data in the study. This was not a conventional cohort study, in that, for example, those in year 2 could not be assumed to be a subset of those in year 1 of the cohorts, with students joining and leaving the study at different stages, mainly dependent on the participation of their host medical school that specific year. However, Table 1 describes the ‘classical’ attrition rates of those who were in the original entry cohorts for 2007 and 2008. Not all medical schools provided academic outcomes on all three areas (i.e. skills, theory and overall result for year (e.g. pass first time)) for each year they participated in the study. At the time of the study, there were 32 medical schools in the UK, of which 26 used the UKCAT, with up to 18 of these providing academic progression data. In 2007–8, approximately 7000 students per year in the UK were admitted to medical school [29]. Thus, our sample of 6812 individuals, with at least one academic outcome recorded, represented roughly half of all UK medical entrants to standard courses during that period. We had no reason to assume that those included in the study sample were significantly different from the overall national medical student population at that time. In this regard, the demographic characteristics of our sample appeared in line with what was known about the medical student population at that time, consisting of 58 % of females, mainly of White ethnicity (70 %) and having an average age of approximately 19.5 years at medical school entry [30].

Table 2 reports the completeness of the data in terms of outcomes. A summary of the sociodemographic characteristics of the sample of medical students is presented in Table 3. As can be seen, just under half the entrants had attended non-selective schools and one in six were over 21 at entry. The entrants were almost exclusively from professional socioeconomic backgrounds. The mean UCAS tariff (calculated for best of three A-levels, best of five Scottish Highers or best of six ILEs, excluding General Studies grades) for the study sample was 351.73 (SD, 15.47). This equates to a point somewhere between AAB and AAA grades at A-level (i.e. most entrants were scoring the maximum potential tariff points). Note that the A* grade at A-level was not in use at the time the study participants applied to university. Those entrants sitting the UKCAT in 2006 had a mean total score of 2482.79 (SD, 217.50) on the test, whilst those sitting in 2007 had a mean score of 2522.70 (SD, 198.21).

**Table 2:** Medical school performance outcomes and proportion of data present for each outcome

Academic Year	Proportion passing first time (%)	At least one exam outcome present	Percentage of theory scores present	Percentage of skills scores present
1	5463/6371 (85.75)	6425/6812 (94.32)	4922/6812 (72.25)	3538/6812 (51.94)
2	5569/6236 (89.30)	6253/6812 (91.79)	4815/6812 (70.68)	4125/6812 (60.55)
3	5067/5524 (91.73)	5533/6812 (81.22)	4135/6812 (60.70)	4074/6812 (59.81)
4	4323/4613 (93.71)	4620/6812 (67.82)	4373/6812 (64.19)	3594/6812 (52.76)
5	2786/2894 (96.27)	2894/6812 (42.48)	2777/6812 (40.77)	2640/6812 (38.76)

**Table 3:** Sociodemographic and educational characteristics of entrants to the participating medical schools

Baseline variable	Proportion (%)	Missing (%)
Male sex	2874/6812 (42.19)	0/6812 (0)
Age ≥ 21 years at entry	1147/6812 (16.84)	0/6812 (0)
Non-selective school attended	3097/5725 (54.10)	1087/6812 (15.96)
Non-white ethnicity	2053/6714 (30.58)	98/6812 (1.44)
Non-professional socioeconomic background	125/5653 (2.21)	1159/6812 (17.01)
Registered as special educational needs for UKCAT	65/6812 (0.95)	0/6812 (0)

According to the relevant UKCAT Technical Reports, the mean score for all medical school applicants sitting the UKCAT was 2407 (SD, 259) in 2006 and 2430 (SD, 255) in 2007 [31, 32]. This meant that the entrants in the study sample scored, on average, 75 points higher than overall applicants in 2006, and 92 points higher in 2007. Thus, a larger difference in UKCAT score between applicants and entrants was observed in the later cohort.

Only a small proportion of entrants (< 1 %) were classified as having SEN status. Exploratory analyses revealed no association between SEN status and academic progression outcomes and so this potential predictor variable was not included in subsequent analyses.

### Univariable analysis

**Fig. 2:**
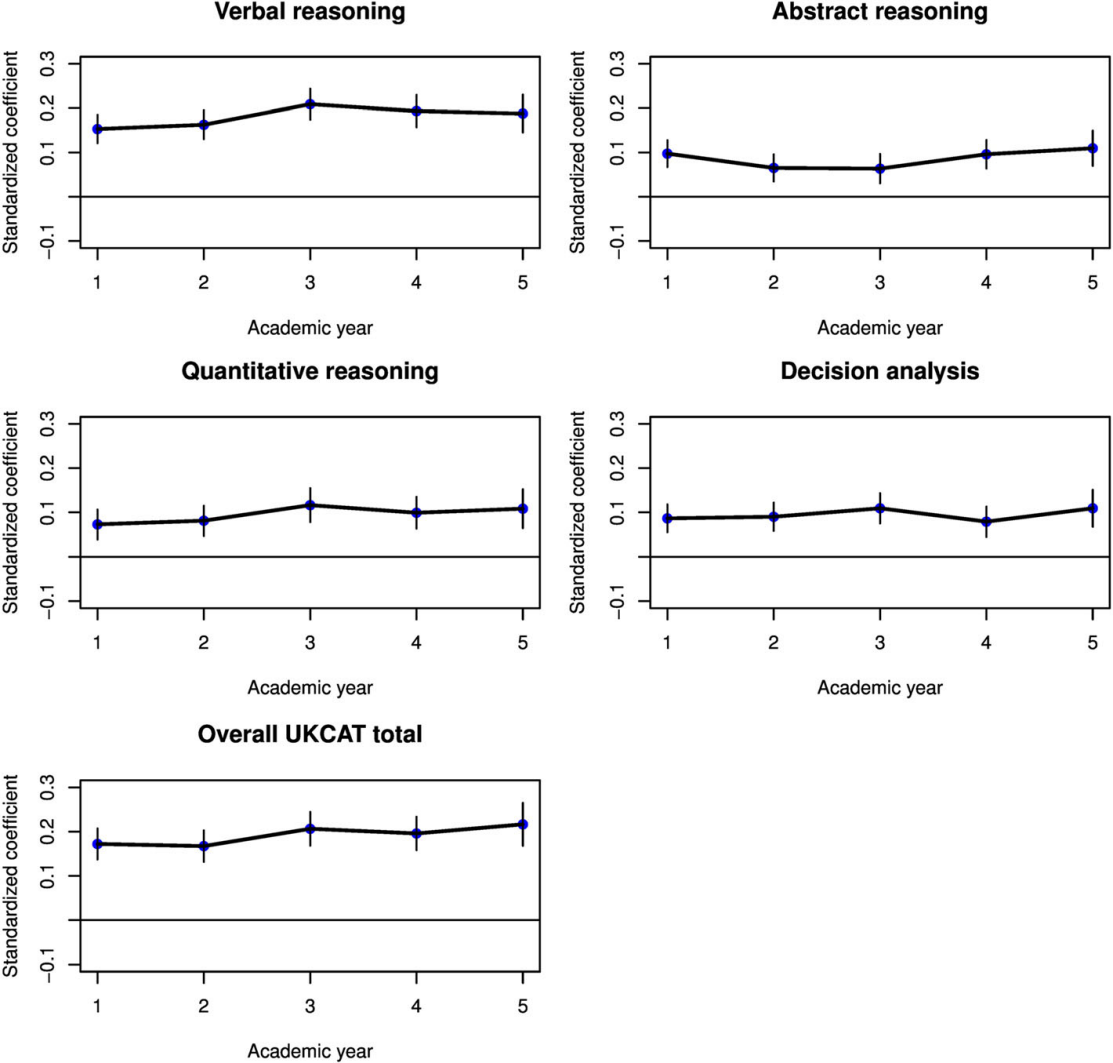
Results from a multilevel univariable regression of performance on theory-based medical school exams on the scales of the UKCAT, with standardised regression coefficients (blue) and associated 95 % confidence intervals plotted for each year of progression at medical school

**Fig. 3:**
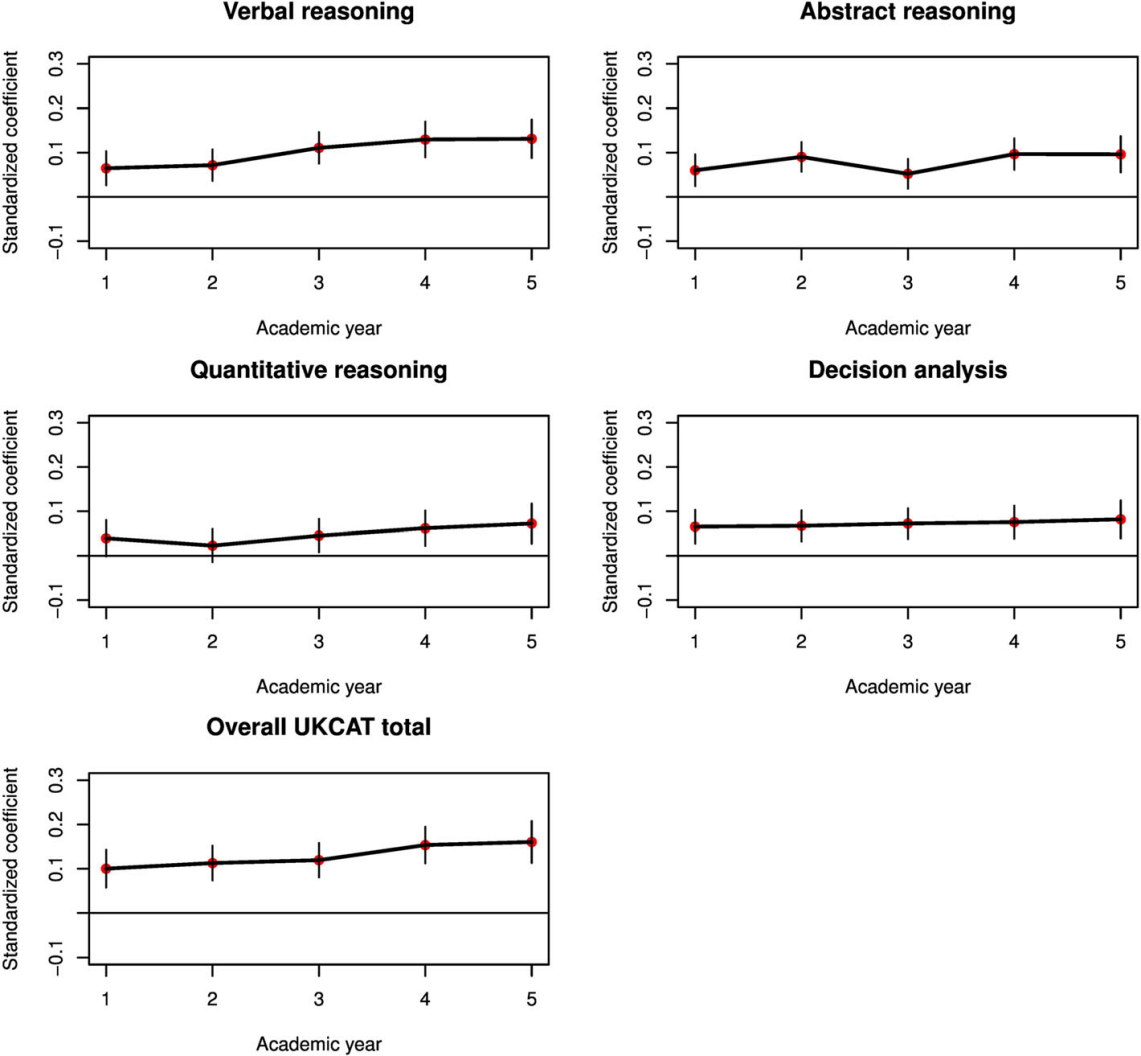
Results from a multilevel univariable regression of performance on skills-based medical school exams on the scales of the UKCAT, with standardised regression coefficients (red) and associated 95 % confidence intervals plotted for each year of progression at medical school

In order to estimate the unadjusted (raw) relationship between the UKCAT scores and medical school undergraduate performance in theory and skills tests, a series of univariable beta (regression) coefficients were derived from linear mixed models. Due to the large number of coefficients, the results are depicted in graphical form in Figs. 2 and 3, according to the specific UKCAT scale score used as a predictor.

All the scales and total scores from the UKCAT significantly predicted performance at theory exams throughout all 5 years of medical school (Fig. 2). The coefficients were generally relatively small, at approximately 0.1 to 0.2. Likewise, the UKCAT scores generally significantly predicted performance at skills-based exams throughout medical school, though ‘quantitative reasoning’ scores were of borderline or non-significance for years 1 and 2 (Fig. 3). Again, absolute values for the coefficients were small, at the 0.05 to 0.1 level. Some trend was noted for the ‘verbal reasoning’ and total UKCAT scores to more strongly predict skills performance as medical school progressed.

**Fig. 4:**
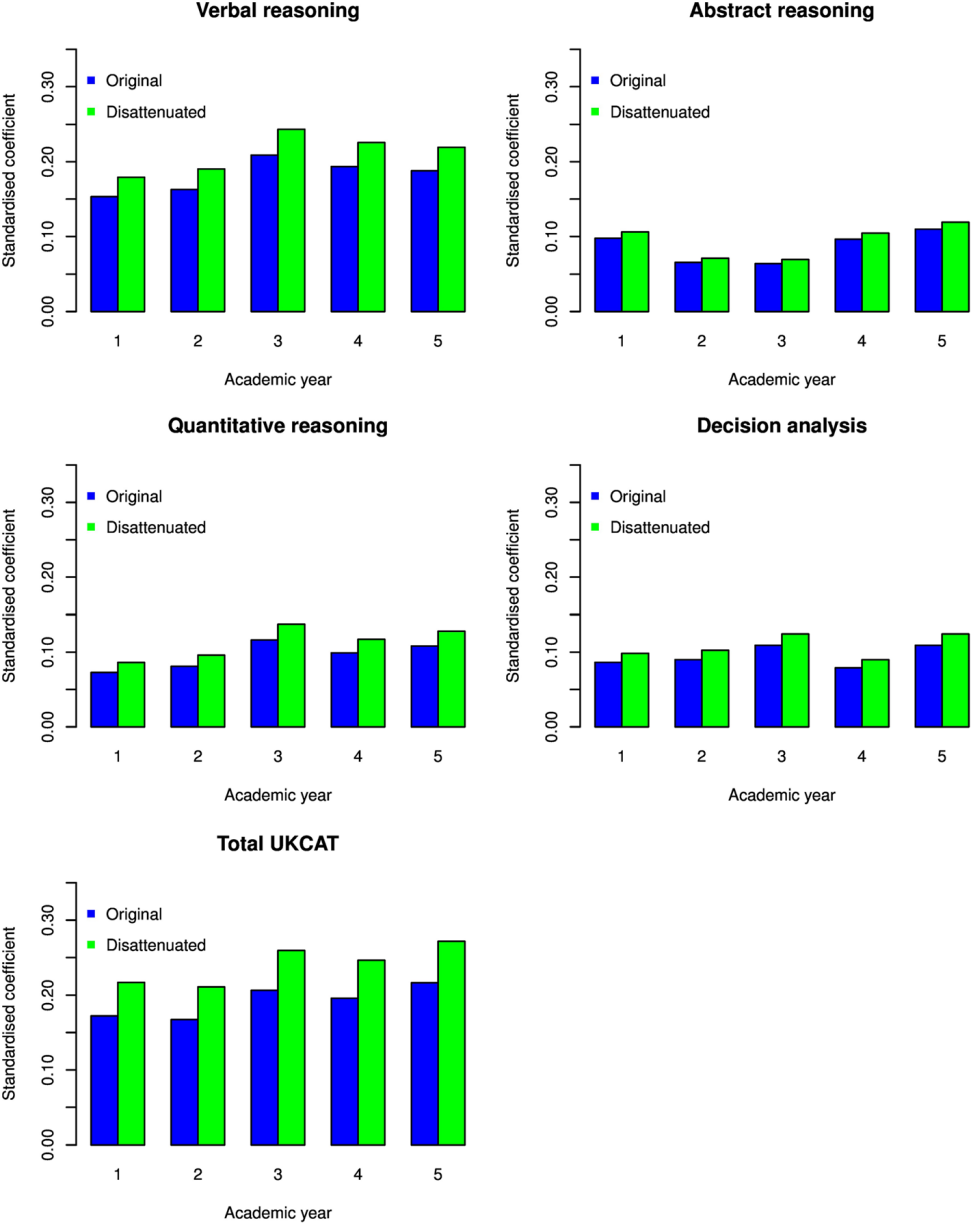
Bar charts depicting regression coefficients for the prediction of theory performance from the UKCAT scores for each year at medical school. The blue bars represent the original coefficients whilst the green bars represent those corrected for the attenuating effects of range restriction

**Fig. 5:**
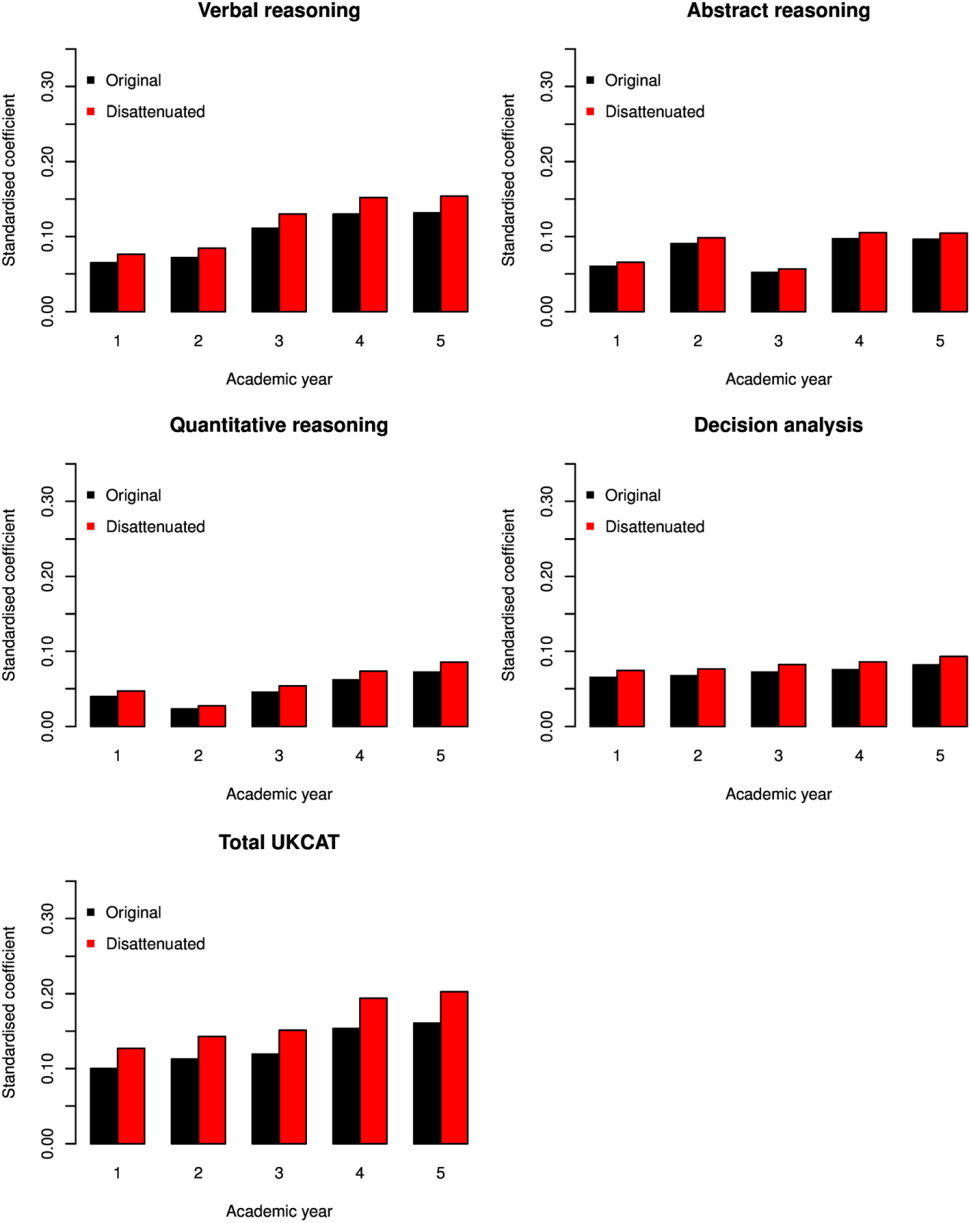
Bar charts depicting regression coefficients for the prediction of skills performance from the UKCAT scores for each year at medical school. The black bars represent the original coefficients whilst the red bars represent those corrected for the attenuating effects of range restriction

It is well recognised that, in selection tests, estimates of the correlation between predictors and outcome are subject to attenuation, mainly due to range restriction (i.e. the outcome can only be observed in those successfully passing a selection test), but also due to imperfect reliability in both the predictor (i.e. UKCAT scores) and the outcome (e.g. theory scores) [33]. Therefore, a correction was applied to the coefficients. The corrected values are depicted in Figs. 4 and 5, alongside uncorrected values. The correction applied in this case is known as ‘Thorndike II’, which corrects for direct range restriction – this is when the range of the predictor is restricted as it is used as a criterion in candidate selection [34]. The magnitude of the increases in the coefficients following correction can thus be observed in Figs. 4 and 5.

The results from the GEE analysis of the odds of passing end of year assessments first time are depicted in Table 4. As can be seen, on univariable analysis, all UKCAT scale scores were positively related to the odds of passing a year at first attempt. For example, for every SD above the mean (for that cohort of UKCAT testees) a student had scored on ‘verbal reasoning’ at application, the odds of passing an end of year exam first time increased by approximately 23 %.

**Table 4:** Results of logistic regression (within a Generalised Estimating Equations (GEE) framework) predicting the odds of passing each year at first attempt (compared to another academic outcome) according to standardised UKCAT scores compared to logistic regression (GEE) of standardised UKCAT scores, adjusted for school advanced qualifications. Both models control for time (i.e. year of sitting)

UKCAT scale	Univariable OR (95 % CI)	*P* value	Adjusted OR (95 % CI)	*P* value
Verbal reasoning	1.23 (1.16–1.30)	< 0.0001	1.19 (1.11–1.28)	< 0.0001
Quantitative reasoning	1.12 (1.06–1.20)	0.0002	1.03 (0.95–1.10)	0.50
Decision analysis	1.18 (1.11–1.25)	< 0.0001	1.10 (1.03–1.17)	0.006
Abstract reasoning	1.19 (1.12–1.25)	< 0.0001	1.12 (1.05–1.19)	0.0009
UKCAT total	1.31 (1.23–1.40)	< 0.0001	1.20 (1.11–1.30)	< 0.0001

### Multivariable analysis

**Fig. 6:**
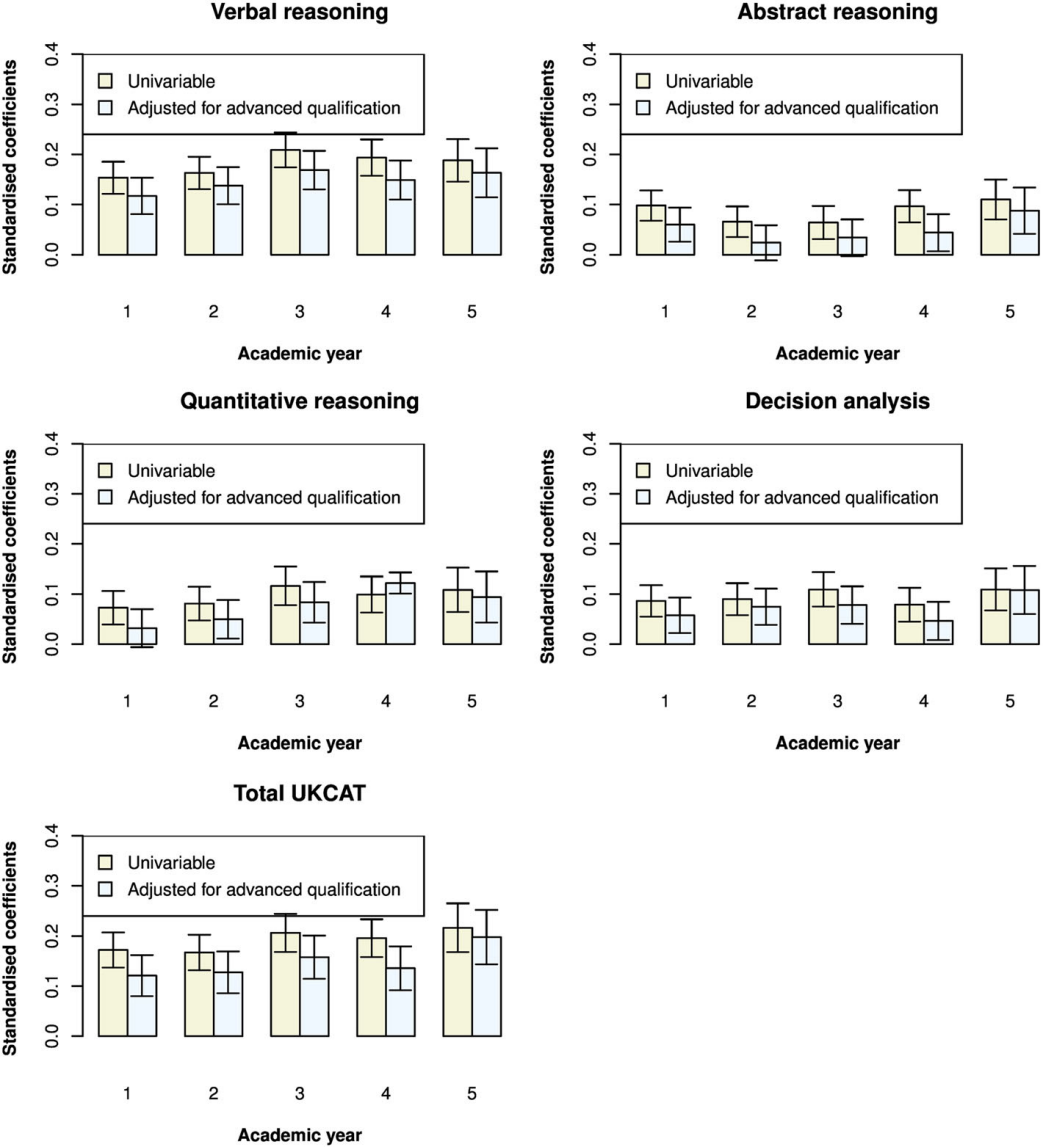
Bar graphs of the standardised regression coefficients from multilevel regressions of performance on medical school theory-based exams on UKCAT scores. Two sets of coefficients are portrayed – those with and without adjustment for advanced qualifications. The whiskers represent the 95 % confidence intervals for the coefficients

**Fig. 7:**
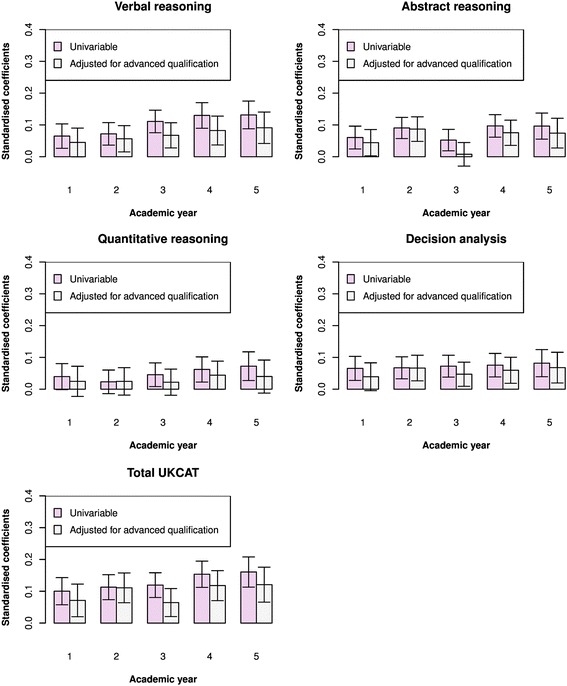
Bar graphs of the standardised regression coefficients from multilevel regressions of performance on medical school skills-based exams on UKCAT scores. Two sets of coefficients are portrayed – those with and without adjustment for advanced qualifications. The whiskers represent the 95 % confidence intervals for the coefficients

In order to evaluate the value that the UKCAT scores add to predicting undergraduate performance, over and above that provided by traditional educational attainment, we provide the results for models adjusting for this covariate only. Figure 6 shows a bar graph of the coefficients from the multilevel univariable model of theory on the UKCAT scales compared to those obtained after adjusting for advanced qualification attainment, prior to medical school. The height of the bar graph represents the magnitude of the coefficients. Likewise, Fig. 7 depicts the coefficients, with and without adjustment for advanced qualification attainment, for the prediction of skills performance in medical school. From Fig. 6, we see that, in all instances, the univariable coefficients of the UKCAT scores for the prediction of theory performance are all positive and statistically significant at the *P* < 0.05 level (as also portrayed in Fig. 2). In all but two cases (‘quantitative reasoning’ for year 4 and ‘decision analysis’ for year 5), their magnitudes reduce upon adjusting for the effect of advanced qualification. In addition, it may be noted that adjusting for advanced qualification renders the coefficient of ‘abstract reasoning’ for year 3 and ‘quantitative reasoning’ for year 1 non-significant. From Fig. 7, we see that, in most instances, the coefficients for the prediction of skills performance by the UKCAT scale scores reduce upon adjusting for the effects of advanced qualification. However, it can be seen that the exception to this is for the prediction of skills score in year 2 of medical school; aside from the UKCAT ‘verbal reasoning’ scale, the coefficients change little after adjustment for advanced qualification attainment.

Models adjusting for all the available confounding variables were also estimated. The results were generally very similar to the models that adjusted for prior educational attainment alone. The results from these multivariable models are depicted in Additional file 1: Supplementary and Technical Appendix (Additional file 1: Figs. S3 and S4).

Table 4 shows the odds of passing each year at first attempt as predicted by the different UKCAT scales from the GEE-based logistic regression after adjusting for the effect of advanced qualification, in addition to time. From the table, we can conclude that the odds of passing each year at first attempt are generally statistically significant, with increasing UKCAT total score associated with the highest increased odds of a pass at first attempt. However, the effect of the ‘quantitative reasoning’ score is less than that of the other UKCAT components and is not statistically significant once the influence of advanced qualifications is accounted for.

### Conceptualising the UKCAT as a screening tool

**Fig. 8:**
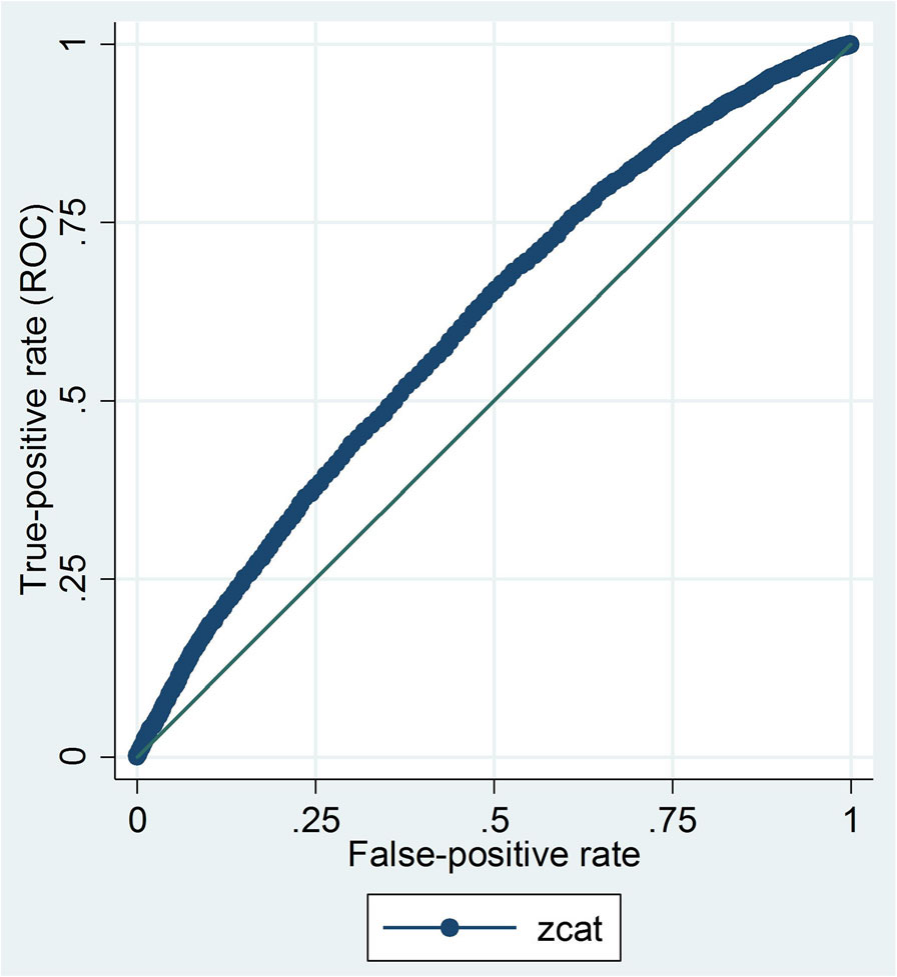
Receiver operator characteristic curve for the use of the total UKCAT as a tool to screen out medical applicants who are likely to fail at least 1 year of medical school at first attempt; in this case, missing outcomes were singly imputed

We can see from Fig. 8 that the ROC curve is rather flat, although the area under the curve (AUC) is, at 0.61, significantly different to 0.5 (95 % confidence intervals 0.60 to 0.61). The ROC curve and the table of sensitivities, specificities and classifications for the various potential cut-points of UKCAT total score were examined (see Table 5 for an abbreviated version of this). From the ROC curve and this table, we selected a standardised UKCAT score of zero (i.e. the mean for that cohort of candidates) as the postulated ‘screening threshold’ to focus on, for illustrative purposes. This is because, a standardised z-score of around zero provides a compromise between sensitivity (i.e. ability of the test to pick up true ‘cases’) and specificity (i.e. the ability to detect true ‘non-cases’). Moreover, the accuracy of classification of applicants drops off fairly steeply after this point; thus, a higher cut-point is likely to be undesirable from this perspective. In addition, to further illustrate the potential impact of utilising the UKCAT as a screening tool, using the real and imputed outcomes, we constructed a two-by-two contingency table for three varying hypothetical cut-off scores (Table 6).

**Table 5:** A table of the estimated characteristics of the UKCAT total score (in this case standardised as a z-score) for screening-out medical school applicants likely to fail at least 1 year of university at first sitting

Cut-off z-score (standardised UKCAT total score)	Sensitivity	Specificity	Accuracy of classification
≤ –3.0	99.9 %	0.2 %	62.6 %
≤ –2.5	99.7 %	1.0 %	62.8 %
≤ –2.0	99.0 %	3.0 %	63.1 %
≤ –1.5	97.2 %	7.00 %	63.4 %
≤ –1.0	92.1 %	15.2 %	63.3 %
≤ –0.5	81.7 %	29.6 %	62.2 %
≤ 0.0	62.1 %	50.5 %	57.8 %
≤ 0.5	37.6 %	72.8 %	50.8 %
≤ 1.0	18.0 %	89.0 %	44.6 %
≤ 1.5	6.7 %	96.2 %	40.2 %
≤ 2.0	1.8 %	99.1 %	38.2 %
≤ 2.5	0.5 %	99.8 %	37.6 %
≤ 3.0	0.1 %	99.9 %	37.5 %

**Table 6:** Two-by-two contingency tables for the UKCAT as a hypothetical screening test for failing to pass, at first sitting, at least 1 year at medical school

Screening result	Fail at least one exam at first sitting		
Screen positive (low threshold: z ≤ –1)	No	Yes	Total
No	14,198	7808	22,006
Yes	1152	1465	2617
Total	15,350	9273	24,623
Screen positive (medium threshold: z ≤ 0)	No	Yes	Total
No	9560	4370	13,930
Yes	5790	4903	10,693
Total	15,350	9273	24,623
Screen positive (high threshold: z ≤ 1)	No	Yes	Total
No	2844	939	3783
Yes	12,506	8334	20,840
Total	15,350	9273	24,623

Three thresholds are set for the standardised UKCAT score: low (z ≤ –1), medium (z ≤ 0), and high (z ≤ 1). Missing values for outcomes (including for unsuccessful applicants) were singly imputed, conditioned on relevant observed variables

From Table 6, we can see that, if the UKCAT score is used as a screening threshold, with z = 0 set at the cut-point for candidate rejection, 46 % (4903/10,693; in effect the positive predictive value of the test) of those who screen positive are estimated to fail at least one of their year’s at first attempt. In contrast, only 31 % (4370/13,930) of those that are above the screening threshold are estimated to fail at least one of their year’s at first attempt. Thus, the absolute risk reduction resulting from the screening process is 15 %. This equates to a ‘number needed to treat’, or rather in this case, ‘number needed to screen’, of seven. This could be viewed as an overall index of the effectiveness of the UKCAT as a selection method. We can also calculate hypothesised values for the UKCAT at this selected ‘screening’ threshold for sensitivity (53 %) and specificity (62 %).

We can also see that, using a mid-range cut-point, in order to reject 4903 candidates who would be likely to fail at least one exam, we would need to reject 5790 candidates who would probably pass all their end of year assessments first time. This ratio (5790:4903) could be termed as the NNR; in this case 1.18 (approximately one, if rounded down). This could be conceptualised as the number of acceptable candidates we would need to reject in order to screen out one ‘poor’ candidate. This number rises to 1.5 if a higher threshold (z ≤ –1, i.e. one standard deviation above the mean) is used. In contrast, the NNR reduces to around 0.8 if a lower threshold is selected (z ≤ 1). However, as can be seen from Table 6, when this lower cut-point is selected, the absolute number of higher risk candidates rejected is fairly low at 1465. Moreover, at this threshold, the estimated specificity of the test is also very low at around 15 % (Table 5).

## Discussion

This study was intended to provide the most comprehensive answer feasible as to whether UKCAT scores predict future performance in undergraduate medical training, and to the extent that it adds value within the selection process. Our findings suggest that the test scores are significantly predictive of most aspects of undergraduate performance. Whilst these effects are not always independent of other, potentially confounding, factors, many of the associations with performance remained statistically significant despite controlling for the influence of prior education attainment. Thus, the test can be assumed to add incremental value above and beyond that provided by actual or predicted A-level (or equivalent) grades. When predicting an overall pass at first sitting for a year at medical school, only the prediction of this outcome from the ‘quantitative reasoning’ score became statistically non-significant once prior educational achievement was controlled for. This may be because such quantitative reasoning skills are already well tested in science-based advanced school qualifications, and thus this particular subscale adds little incremental predictive value in this respect.

Whilst the absolute ability of the UKCAT to predict medical school performance appears modest, the challenges of establishing the true ‘construct-level’ for such a selection test cannot be underestimated. McManus et al. [19] elegantly outline these issues, which include the attenuating effects of restriction of range, imperfect test reliability and the homogeneity amongst candidates both in terms of predictors and outcomes. In order to address these problems, firstly we used a formula to ‘disattenuate’ the regression coefficients from our univariable analyses. Secondly, we took a novel approach, using data imputation, to simulate being able to observe missing outcomes in the UKCAT candidates. The findings suggest that, even using the test as a sole selection test, use of the UKCAT as a threshold for application decisions may result in reduced academic failure rates in medical school. Moreover, this is the first study, to our knowledge, that introduces a more pragmatic approach to understanding the potential practical implications of aptitude testing via our NNR estimate.

Our results are largely in line with previous published findings. Unsurprisingly (given that some of the participants are shared between the studies), our observations in relation to performance in year 1 of medical school are almost identical to those cited by the UKCAT12 study [13]. Our univariable findings are also broadly consistent with previous, local studies that have observed some ability of the UKCAT scores to predict aspects of undergraduate performance into the latter, clinical years of training [14, 15]. However, in contrast to the MCAT, the UKCAT scales at times seem to have increased independent predictive validity as medical school education progresses into the final clinical years [17]. This is possibly due to the MCAT having a substantial knowledge-testing component, which becomes less relevant to predicting academic outcomes as undergraduate medical education progresses. This is in contrast to the UKCAT, which does not evaluate semantic knowledge as such. The trend for predictive ability to persist or increase is particularly observed for the ability of ‘quantitative reasoning’ and total score to predict theory assessment performance once confounders are adjusted for. It may reflect the relative importance of cognitive ability over traditional educational attainment as the effects of previous schooling decays. In line with previous findings, we observed that better performance at medical school assessments was generally associated with female sex, older age at entry, attendance at a non-selective state school, White ethnicity, and better A-level (or equivalent) grades.

### Strengths and potential limitations of the study

This is the first national study to assess the predictive validity of the UKCAT throughout the entirety of medical undergraduate education. The large number of universities and participants provides statistical power to this study, as well as increasing the likelihood that the findings are generalizable to UK medical schools. Indeed, this is the first national study to investigate the predictive ability of the UKCAT into the clinical years of training, whilst controlling for the effects of a number of potentially confounding factors.

Nevertheless, a number of potential limitations must be highlighted. Firstly, in terms of the outcome measures, skills and theory based assessments were not operationally defined, and therefore rely on the participating medical schools to categorise the evaluations accordingly. It is reasonable to assume that assessments categorised as ‘theory’ evaluated knowledge required of the undergraduate curricula. However, the nature of skills assessments may have varied to a relatively greater degree across medical schools, although these may have included Objective Structured Clinical Examinations or similar. Overall, the relationship between skills performance and UKCAT scores was weaker than for theory exams. It could be speculated that this relationship may have been even less marked if medical schools had only categorised summative evaluations with a strong focus on procedural knowledge and interpersonal functioning (e.g. observed role plays, etc.) as skills. Thus, it is possible that the association between skills and UKCAT scores was inflated by the inclusion, by some medical schools, of assessments that relied on traditional cognitive ability and semantic knowledge. Nevertheless, to some extent, the variability between medical schools (and across time) in the nature and standards of both theory and skills assessments would have been dealt with by the standardisation of the scores within both institutions and cohorts.

It should also be noted that, for this study, the most recent UKCAT scores were used as the primary predictor. These may not have been the best metric of ability, though by the use of these we eliminated ‘practice runs’ and also based the analysis on the scores on which admission decisions are based.

The number of participating universities varied from year to year, and missingness related to this was probably due to chance (hence missing completely at random; it may have been mainly due to medical schools failing to return outcome results). Further, it should be noted that this was not a classical cohort study as subsequent years were not a subset of the original entry cohorts (although we provide the values for this ‘conventional’ attrition rate in Table 1), with whether participants joined or left the study being mainly dependent on their medical school participating that specific year. Sensitivity analyses were conducted to evaluate the potential effects of missing data on the results. We subsequently observed that the results from imputed and non-imputed datasets differed for later years and, therefore, some caution must be exercised when making inferences. However, methodological research supports the use of multiple imputation through chained equations where the pattern of missingness is arbitrary. Therefore, unless a substantial portion of the missing data was non-ignorable, the results from the imputed datasets should be relatively trustworthy [25]. Thus, where the results differ it may be those from the imputed datasets that are more reliable. There is also some uncertainty that must be accepted about the ‘construct-level’ validity of the UKCAT due to the attenuation effects apparent in selection tests [19]. As mentioned above, we were able to crudely correct for this using the ‘Thorndike II’ method in this situation [34]. However, this approach assumes direct range restriction only (i.e. selection was based only on the UKCAT scores) and this is not the case in reality. Moreover, our attempts to estimate the NNR value for the UKCAT as a screener using single imputation could be viewed as based on the (strong) assumption that unobserved outcomes are related to observed values in the same way as non-missing outcomes. Nevertheless, we consider this exploratory analysis as important in beginning to understand the practical implications for the use of the UKCAT within the context of medical selection, where candidate variance is low, poor academic outcomes uncommon, but the competition ratio is high.

Attempts have been made to equate the UKCAT scores in order to ensure that the results are comparable across time [35]. However, significant shifts in the score over time suggest that test equating has not been entirely achieved, possibly due to differences in actual performance of subsequent cohorts (e.g. later cohorts would have access to increased practice opportunities and material). Thus, the properties of the UKCAT may have changed to some extent over time and it is not clear to what extent our findings apply to subsequent cohorts.

### Implications for practice and policy

Previous research suggests that universities that use the UKCAT scores as a threshold for interview or place offer may reduce the level of disadvantage faced by certain under-represented groups of applicants, compared to those using the test in a different mode [7]. Moreover, the UKCAT may be less sensitive to the school type-attended (e.g. selective versus non-selective) compared to school leaving qualifications or predicted grades [8]. This is especially important given that there is emerging evidence that the overall performance of a candidate’s secondary school may be inversely related to an individual’s later achievement in higher education, including in medical school [13]. In this case, the total score appeared to be the element of the UKCAT that was the best predictor of a future entrant’s performance, as it will reflect performance on all the constituent scales. Our findings thus confirm that universities wishing to widen participation may wish to use the UKCAT as a relatively strong component of the selection process as it will have some ability to predict academic performance whilst not furthering the disadvantaging of candidates from certain under-represented groups. Moreover, in contrast to the UKCAT, the Biomedical Admissions Test, used in medical selection by some universities, has been unable to demonstrate any incremental predictive validity, over and above conventional measures of knowledge or educational attainment [36]. This should be considered when institutions are considering selection processes.

However, despite having four subscales, the UKCAT may best be conceptualised as testing two main dimensions of cognitive functioning, namely verbal and non-verbal reasoning [10]. Thus, the total score (consisting of three non-verbal and only one verbal scale score) may put too much emphasis on non-verbal performance. Rescoring so that an average of the non-verbal scales is combined with the verbal reasoning score may be a fairer way to obtain a more balanced metric of ability.

Although the magnitude of the effect of UKCAT scores on performance was relatively small, our estimates of NNR suggest some considerable practical utility of the UKCAT as a tool in helping to select out candidates more likely to require at least one resit at medical school. The NNR value of 1.18 (when a cut-off representing the average UKCAT total score for applicants was used), though derived relatively crudely, also suggests that, in a highly competitive selection process, the use of the UKCAT as a ‘screening tool’ for subsequent academic performance may be acceptable. We also reported the likely impact of having either relatively high or low thresholds for the UKCAT, when using the test in this manner. Indeed, across medical schools and time, a variety of cut-points have been used by universities in the UKCAT consortium who use the test scores in this manner, mainly to guide the decision about whether to invite a candidate for interview. However, the median scores used as a threshold by institutions have tended to rise over time, and tend to be slightly above the average score obtained by applicants sitting the test [20]. Our findings suggest that the use of higher thresholds may reduce the risk of future adverse academic outcomes in students further, but at the cost of rejecting a higher ratio of candidates who would have been likely to have done well. Conversely, lower score thresholds reduce the risk of rejecting this latter group of candidates, but will increase the risk that applicants are admitted who are at higher risk of later academic problems. Thus, the choice of threshold would be a subjective one, decided on by medical school admissions teams. No doubt the competition ratio that a specific university encounters would play a role in such decision making; those with more fierce competition for places may be tempted to set a higher threshold, with the opportunity of identifying candidates less likely to do well academically appearing to offset the attendant risks of rejecting acceptable candidates. Thus, we can see that, in the context of medical school applications, where the competition ratio at individual medical schools is approximately 11:1, an NNR of roughly one may be acceptable to admissions teams (though possibly not to candidates), especially given the direct and indirect costs of resits and failures to progress. However, it should be highlighted that selection tests such as the UKCAT are not intended to be used in isolation but in conjunction with other selection criteria. Thus, the estimated NNR in this case only reflects the effectiveness of the UKCAT when used alone, rather than in conjunction with other selection approaches. It may be that the use of other selection criteria (such as performance in multiple mini interviews) would further reduce the value to one that reflects a greater utility in selection. Further, there may be genuine uncertainty over the eventual predictive validity of certain selection tests. Thus, it may be that an approach based on Bayesian principles may be useful. Bayes theorem allows us to increase the accuracy of our probabilistic predictions by conditioning our new observations on previous data or knowledge. The approach also allows us to adjust for uncertainty of how applicable our previous knowledge is to the current issue. Thus, a Bayesian framework may eventually allow us to estimate the impact of combining a variety of selection tests in the admissions process, even allowing for our uncertainty regarding predictive validity.

Given the high stakes involved in deciding on how to allocate medical school places, an alternative approach to ‘front-loading’ selection processes would be to admit a larger number of students but have a lower threshold for failing them after evaluating them during the first year. This, however, could also be viewed as costly given the investment made in educating each student during the initial year of undergraduate study. There will also be costs associated with employing poorly motivated doctors at risk of low morale and burnout [37].

## Conclusions

This study has focussed on the cognitive test components of the UKCAT and the relationship between scores and future undergraduate academic performance. However, the recent introduction of Situational Judgment Testing (based on assessing the responses of candidates to presented situations that challenge professionalism) has introduced an element of evaluating the personal, non-academic, qualities of applicants. It is likely that the addition or modification of further cognitive testing is unlikely to add further value into the selection process and further development of the UKCAT should focus on these other, personal attributes, attempting to link them with future performance, especially in post-graduate practice. The order in which selection tests are considered as well as the weighting on each element will also determine the demographics of the selected population. It may be that placing more emphasis on non-academic tests, such as Situational Judgment Tests, or placing them earlier on in the selection filter, will have a positive impact on widening access to medicine [38]. Moreover, as part of a movement towards evidence-based selection, the economic implications of different selection approaches should be evaluated, taking into account both direct costs (e.g. those related to hosting resits) and indirect costs (e.g. those related to future professional misconduct). This is likely to be an important area for future selection focussed research.

## Additional file

Additional file 1: Supplementary and Technical Appendix. (DOCX 841 kb)
