# Supplementary material for: Predictive validity of the UKCAT for medical school undergraduate performance: a national prospective cohort study
Source: BMC Med. 2016 Sep 26;14:140. doi: 10.1186/s12916-016-0682-7 (PMC5026770; doi:10.1186/s12916-016-0682-7)
Supplement: Additional file 1: — Supplementary and Technical Appendix. (DOCX 841 kb) [file 12916_2016_682_MOESM1_ESM.docx]

**Supplementary and Technical Appendix to:**

**Predictive validity of UKCAT for medical school undergraduate performance: a national prospective cohort study**

Paul A Tiffin, Lazaro M Mwandigha, Lewis W Paton, H Hesselgreaves, John C McLachlan, Gabrielle Finn & Adetayo S Kasim

This technical and supplementary appendix contains information on missing data, including a series of sensitivity analyses in relation to this. This appendix also contains figures relating to the results from the analyses relating to continuous outcomes (i.e. *theory* and *skills* scores) and tables relating to the full results from imputed and non-imputed datasets for dichotomous outcomes (i.e. *passing a year at medical school* *at first attempt* vs *another academic outcome*).

**Missingness pattern**

Below are supplementary tables that describe the missingness patterns in the outcome variables and covariates (supplementary tables S1 to S4).

From supplementary table S1, it can be seen that about 61% of the observations in the data had complete information for the covariates. The most frequently occurring missing profile was for observations with data on all covariates but missing data on social demographic background while the least occurring missing profile was for observations with data on all covariates but missing data on advanced qualification and non-white ethnicity.

From supplementary table S2, it can be seen that only 23.18% of the observations had complete data for the outcome (*theory* scores), with 44.91% having monotone missingness. The most occurring monotone missingness profile had drop out at the fifth year while the least frequently occurring monotone missingness profile had data only for first year *theory scores.* There were 19 different arbitrary missingness profiles with the most commonly occurring profile having data available for years 4 and 5 only.

From supplementary table S3, it was noted that complete information for the outcome *skills score* was available for only about 20% of the data, 37.78 % of the missingness was monotone with the most commonly occurring monotone missingness profile having no data across the 5 years of medical school. There were 21 different arbitrary missingness profiles for the *skills score* with the most commonly occurring having data only for year 5 of medical school.

Supplementary table S4 shows that the outcome *passing each year* had the highest percentage of complete data of all the outcomes at 39.97%. There were 48.75% observations with monotone missingness with the most frequently occurring profile having data missing for year 5 of medical school only. Of the 17 different occurring arbitrary missingness profiles, the most commonly occurring one had data missing for years 3 and 5 of medical school. Of the three outcomes *theory, skills* and *pass* *first time*, only the *theory* outcome exhibited declining numbers of responses throughout the 5 years.

| Group | Non  White | Male  sex | Non  selective  school | Social  economic  background | SEN*  For  UKCAT | Age | Advanced  qualification | Count | % |
| --- | --- | --- | --- | --- | --- | --- | --- | --- | --- |
|  |  |  |  |  |  |  |  |  |  |
| 1 | X | X | X | X | X | X | X | 4,135 | 60.70 |
| 2 | X | X | X | X | X | X | . | 608 | 8.93 |
| 3 | X | X | X | . | X | X | X | 762 | 11.19 |
| 4 | X | X | X | . | X | X | . | 141 | 2.07 |
| 5 | X | X | . | X | X | X | X | 287 | 4.21 |
| 6 | X | X | . | X | X | X | . | 601 | 8.82 |
| 7 | X | X | . | . | X | X | X | 53 | 0.78 |
| 8 | X | X | . | . | X | X | . | 127 | 1.86 |
| 9 | . | X | X | X | X | X | X | 14 | 0.21 |
| 10 | . | X | X | X | X | X | . | 1 | 0.01 |
| 11 | . | X | X | . | X | X | X | 57 | 0.84 |
| 12 | . | X | X | . | X | X | . | 7 | 0.10 |
| 13 | . | X | . | X | X | X | X | 3 | 0.04 |
| 14 | . | X | . | X | X | X | . | 4 | 0.06 |
| 15 | . | X | . | . | X | X | X | 3 | 0.04 |
| 16 | . | X | . | . | X | X | . | 9 | 0.13 |
| Total |  |  |  |  |  |  |  | 6,812 | 100 |

Supplementary table S1. Missingness patterns in the covariates. Each ‘X’ represents each instance where data are present (i.e. the first row represents the proportion of cases with no missing data).

*Classified as having Special Educational Needs for the purposes of the UKCAT

| Group | Year 1 | Year 2 | Year 3 | Year 4 | Year 5 | Count | % |
| --- | --- | --- | --- | --- | --- | --- | --- |
|  |  |  |  |  |  |  |  |
| Completers | | | | | | | |
| 1 | X | X | X | X | X | 1,579 | 23.18 |
| Monotone Missingness | | | | | | | |
| 2 | X | X | X | X | . | 1,035 | 15.19 |
| 3 | X | X | X | . | . | 870 | 12.77 |
| 4 | X | X | . | . | . | 549 | 8.06 |
| 5 | X | . | . | . | . | 133 | 1.95 |
| 6 | . | . | . | . | . | 473 | 6.94 |
| Arbitrary Missingness | | | | | | | |
| 7 | X | X | X | . | X | 111 | 1.63 |
| 8 | X | X | . | X | X | 351 | 5.15 |
| 9 | X | X | . | X | . | 265 | 3.89 |
| 10 | X | X | . | . | X | 2 | 0.03 |
| 11 | X | . | X | X | X | 6 | 0.09 |
| 12 | X | . | X | X | . | 3 | 0.04 |
| 13 | X | . | X | . | . | 17 | 0.25 |
| 14 | X | . | . | . | X | 1 | 0.01 |
| 15 | . | X | X | X | X | 4 | 0.06 |
| 16 | . | X | X | X | . | 14 | 0.21 |
| 17 | . | X | X | . | . | 9 | 0.13 |
| 18 | . | X | . | X | X | 13 | 0.19 |
| 19 | . | X | . | . | . | 13 | 0.19 |
| 20 | . | . | X | X | X | 1 | 0.01 |
| 21 | . | . | X | X | . | 458 | 6.72 |
| 22 | . | . | X | . | . | 28 | 0.41 |
| 23 | . | . | . | X | X | 476 | 6.99 |
| 24 | . | . | . | X | . | 168 | 2.47 |
| 25 | . | . | . | . | X | 233 | 3.42 |
| Total |  |  |  |  |  | 6,812 | 100 |

Supplementary table S2. Missingness patterns for *theory* scores. Each ‘X’ represents each instance where data are present (i.e. the first row represents the proportion of cases with no missing data). Patterns are categorised as either monotone (i.e. where data relating to all subsequent years are missing after the initial missing data year) or arbitrary (i.e. non-monotone).

| Group | Year 1 | Year 2 | Year 3 | Year 4 | Year 5 | Count | % |
| --- | --- | --- | --- | --- | --- | --- | --- |
|  |  |  |  |  |  |  |  |
| Completers | | | | | | | |
| 1 | X | X | X | X | X | 1,338 | 19.64 |
| Monotone Missingness | | | | | | | |
| 2 | X | X | X | X | . | 672 | 9.86 |
| 3 | X | X | X | . | . | 413 | 6.06 |
| 4 | X | X | . | . | . | 671 | 9.85 |
| 5 | X | . | . | . | . | 99 | 1.45 |
| 6 | . | . | . | . | . | 699 | 10.26 |
| Arbitrary Missingness | | | | | | | |
| 7 | X | X | X | . | X | 110 | 1.61 |
| 8 | X | X | . | X | X | 2 | 0.03 |
| 9 | X | X | . | X | . | 205 | 3.01 |
| 10 | X | X | . | . | X | 3 | 0.04 |
| 11 | X | . | X | X | . | 3 | 0.04 |
| 12 | X | . | X | . | . | 15 | 0.22 |
| 13 | X | . | . | X | X | 5 | 0.07 |
| 14 | X | . | . | X | . | 1 | 0.01 |
| 15 | X | . | . | . | X | 1 | 0.01 |
| 16 | . | X | X | X | X | 106 | 1.56 |
| 17 | . | X | X | X | . | 193 | 2.83 |
| 18 | . | X | X | . | . | 107 | 1.57 |
| 19 | . | X | . | X | . | 77 | 1.13 |
| 20 | . | X | . | . | . | 228 | 3.35 |
| 21 | . | . | X | X | X | 407 | 5.97 |
| 22 | . | . | X | X | . | 357 | 5.24 |
| 23 | . | . | X | . | X | 2 | 0.03 |
| 24 | . | . | X | . | . | 351 | 5.15 |
| 25 | . | . | . | X | X | 147 | 2.16 |
| 26 | . | . | . | X | . | 81 | 1.19 |
| 27 | . | . | . | . | X | 519 | 7.62 |
| Total |  |  |  |  |  | 6812 | 100 |

Supplementary table S3. Missingness patterns for *skills* scores. Each ‘X’ represents each instance where data are present (i.e. the first row represents the proportion of cases with no missing data). Patterns are categorised as either monotone (i.e. where data relating to all subsequent years are missing after the initial missing data year) or arbitrary (i.e. non-monotone).

| Group | Year 1 | Year 2 | Year 3 | Year 4 | Year 5 | Count | % |
| --- | --- | --- | --- | --- | --- | --- | --- |
|  |  |  |  |  |  |  |  |
| Completers | | | | | | | |
| 1 | X | X | X | X | X | 2,723 | 39.97 |
| Monotone Missingness | | | | | | | |
| 2 | X | X | X | X | X | . | 1,280 |
| 3 | X | X | X | X | . | . | 1,086 |
| 4 | X | X | X | . | . | . | 748 |
| 5 | X | X | . | . | . | . | 153 |
| 6 | X | . | . | . | . | . | 54 |
| Arbitrary Missingness | | | | | | | |
| 7 | X | X | X | X | . | X | 113 |
| 8 | X | X | X | . | X | X | 3 |
| 9 | X | X | X | . | X | . | 231 |
| 10 | X | X | . | X | X | X | 6 |
| 11 | X | X | . | X | X | . | 9 |
| 12 | X | X | . | X | . | . | 14 |
| 13 | X | X | . | . | X | X | 4 |
| 14 | X | X | . | . | X | . | 1 |
| 15 | X | . | X | X | X | X | 17 |
| 16 | X | . | X | X | X | . | 14 |
| 17 | X | . | X | X | . | . | 5 |
| 18 | X | . | X | . | X | . | 1 |
| 19 | X | . | X | . | . | . | 15 |
| 20 | X | . | . | X | X | X | 28 |
| 21 | X | . | . | X | X | . | 218 |
| 22 | X | . | . | X | . | . | 11 |
| 23 | X | . | . | . | X | . | 78 |
| Total |  |  |  |  |  | 6,812 | 100 |

Supplementary table S4. Missingness patterns for passing each year. Each ‘X’ represents each instance where data are present (i.e. the first row represents the proportion of cases with no missing data). Patterns are categorised as either monotone (i.e. where data relating to all subsequent years are missing after the initial missing data year) or arbitrary (i.e. non-monotone).

**Sensitivity analyses for missing data**

Under the selection model framework, missing data mechanisms consists of MCAR (Missing Completely At Random), MAR (Missing At Random) and MNAR (Missing- Not At Random). Under MCAR, the assumption is that conditioning on the covariates of interest, the missing mechanism is independent of the observed or unobserved outcomes. Under MAR, conditioning on the covariates, the missing mechanism depends on the observed outcomes but not on unobserved outcomes ^2^. Finally, under the MAR conditioning on the covariates, the missing mechanism depends on the outcomes both observed and unobserved. In reality, the MCAR mechanism is very restrictive so most modelling frameworks assume MAR. To see the impact of missing data on results, the recommended practice is to conduct sensitivity analysis to determine if the MAR assumption is plausible. If not, then the missing mechanism is proved to be MAR which therefore necessitates the missing mechanism to be modelled in addition to parameter estimation that may be of interest.^1, 2^

The *theory* and *skills* scores*, as* outcomes, were modelled using a multilevel model framework by use of a mixed effects model. Since mixed effects models are likelihood based, the assumption of MAR is assumed by invoking ignorability. In order to check whether this assumption was satisfied*,* sensitivity analysis was done by use of Multiple Imputation (MI). The basis of this is multiple datasets are produced where the missing values are replaced with a set of plausible values drawn from particular distributions. Typically the choice of imputation method will depend on the missingness pattern (i.e. is it monotone for the outcome over the entire 5 year period in medical school?) and the nature of the variable being imputed (i.e. is it plausible to assume normality?). There are a wide range of methods available depending on missingness pattern and the distributional assumption of the variable being imputed. Since the continuous outcomes, *theory* and *skills,* were right truncated the assumption of normality for imputation was not justifiable. The missingness pattern for the outcomes for the 5 year period in medical school were also not monotone. Under these circumstances, the most preferred method of multiple imputation is *chained equations* also known as *full conditional specification*. Multiple imputation is done a finite number of times, to determine the number of times this was to be conducted, it was decided to imputed datasets in multiples of 10 ( that is 10,20,30,…) up to the point where the results of the MI stabilised (the results of the models pooled from the MI datasets remained unchanged). The optimum number of imputed datasets was to be above 20 (there were no changes in results from the 20 and 30 imputed datasets). So the results from 30 imputed datasets were used for sensitivity analysis.


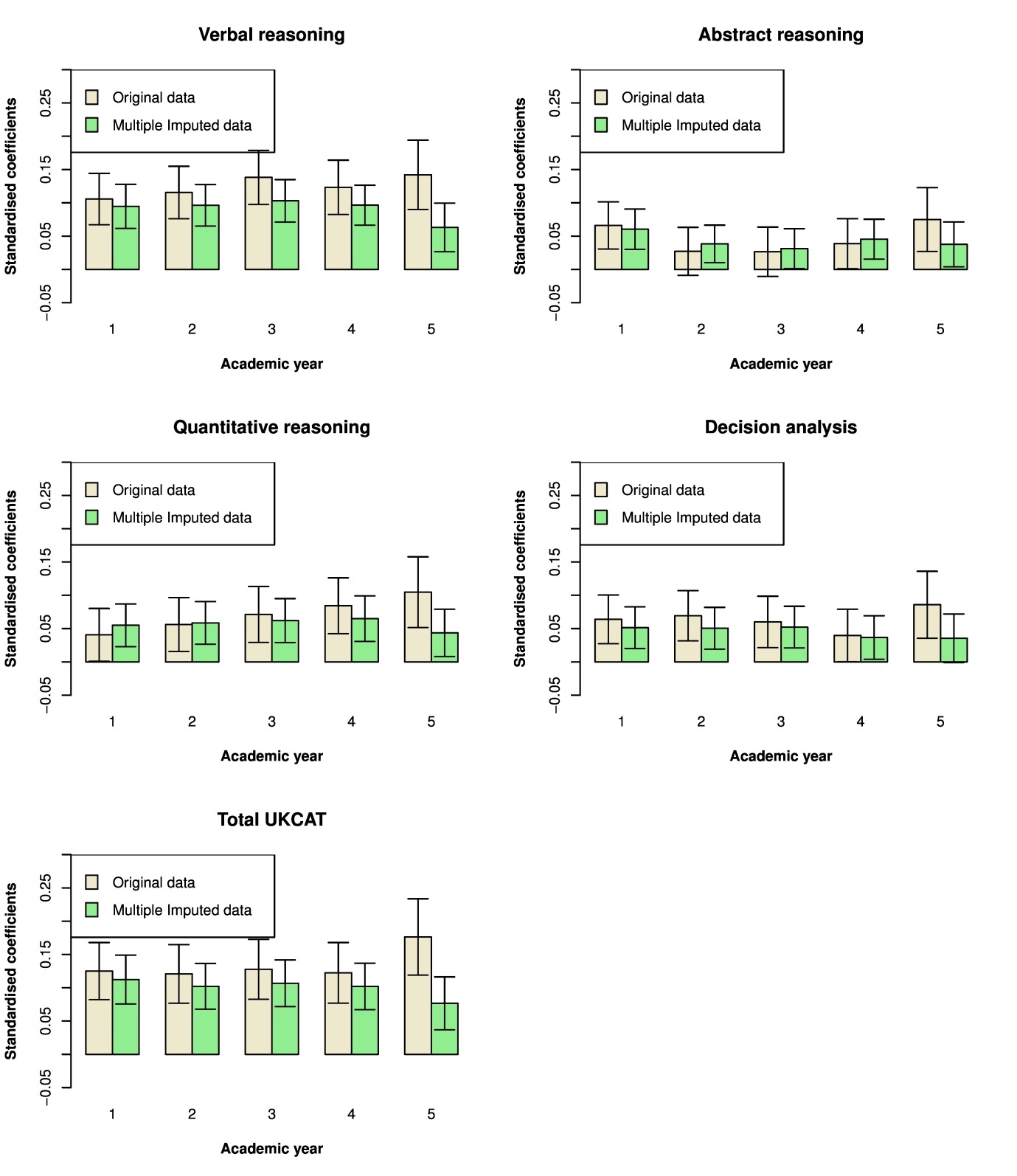


Supplementary figure 1. Bar graphs of standardised regression coefficients from multilevel multivariable regression of performance on *theory* based medical school exams on the scales of the UKCAT for original and multiple imputed data together with plots of their associated 95% confidence intervals.

Supplementary figure 1 depicts bar graphs of the results from the multilevel multivariable regression of performance on *theory* on the different scale scores of the UKCAT. The height of the bar graph represents the magnitude of the coefficient of the UKCAT scales from the model for both the original and imputed datasets. In addition, their respective confidence intervals are plotted for each coefficient. It can be seen that, in all cases, the coefficients from both the original and multiple data are positive. Also, it is noted that in every case, the coefficients from the original data are larger than the coefficients from the imputed data. We assessed the differences in inference regarding the significance of the coefficients from original and imputed data by use of plotted confidence intervals. We observed a difference in inference in six of the 25 models fitted, namely for the *abstract reasoning* (years 2, 3 and 4), *quantitative reasoning* (year 1) and *decision analysis* scale scores (years 4 and 5). This implies that in those instances, the MAR assumption from the multilevel multivariable model is not satisfied and thus the imputed results carry more weight (note that the MAR assumption is satisfied under the MI approach). The conclusion is for the most part, the missing data do not affect the results for the *theory* outcome.


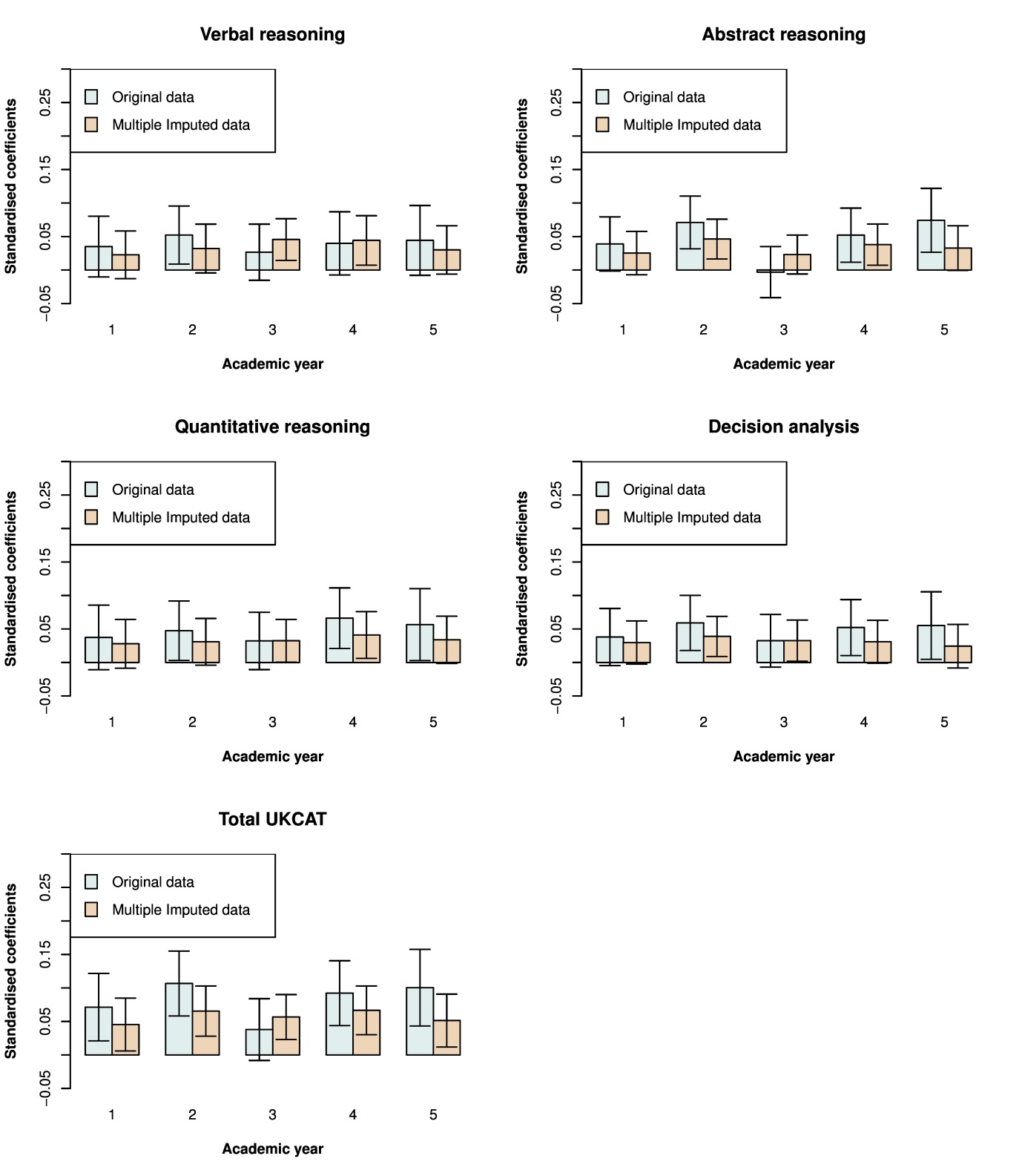


Supplementary figure 2. Bar graphs of standardised regression coefficients from multilevel multivariable regression of performance on *skills* based medical school exams on the scales of the UKCAT for original and multiple imputed data together with plots of their associated 95% confidence intervals.

*Supplementary figure 2* depicts bar graphs of the results from the multilevel multivariable regression of performance on *skills* on the scales of the UKCAT. As in the case of *theory* performance*,* the height of the bar graph represents the magnitude of the coefficient of the UKCAT scales from the model for both the original data and the imputed data. In addition, their respective confidence intervals are plotted for each coefficient. Again, it can be seen that, in all cases, the coefficients from both the original and imputed data are positive. Also, in 19 of the 25 cases, the coefficients from the original data are larger than the coefficients from the imputed data. In the case of *skills*, there is a difference in inference in 11 of the 25 models fitted. That is for *verbal reasoning* (years 2, 3 and 4), *abstract reasoning* (year 5), *quantitative reasoning* (years 2, 3 and 5), *decision analysis* (years 3, 4 and 5) and *total UKCAT* *score* (year 3). This implies that in those instances, the MAR assumption from the multilevel multivariable model is not satisfied and thus the imputed results carry more weight since MI is considered to be valid under MAR. Compared to the *theory* outcome, the data relating to the *skills* outcome are more affected by missingness and thus the results from the multivariable multilevel model ought to be interpreted with caution.

The outcome *passing each year at first attempt* was analysed using multivariable logistic regression (within a Generalised Estimating Equations framework). The GEE approach is considered the preferred choice for modelling multilevel (clustered) data for several types of outcomes (binary, nominal, continuous etc.) in instances where the interest is population average modelling of the data. The drawback with GEE is that in instances where data are missing it is valid only under the MCAR framework. In order to use GEE with a MAR assumption normally two options are available; the first one involves fitting a Weighted GEE (WGEE) where observations from clusters with most data contribute the most to the analysis. This however is only viable in instances where the missingness pattern is monotone. The second option, which does not depend on the missingness pattern being monotone, is MI GEE which involves conducting multiple imputation prior to a GEE analysis.

Since the data had non-monotone missingness for the outcome, *passing each year at first attempt,* MI was conducted by *chained equations.* The number of optimum imputation was decided as in the case of *theory* and *skills* outcome with the optimum number of imputed datasets used being 30. Supplementary tables 5 to 9 show the results of multivariable GEE (prior to MI) and MI GEE. It can be seen that none of the UKCAT scale have significant odds of predicting *passing each year at first attempt* assuming MCAR framework. The MI GEE results under a MAR assumption show that the resulting odds of *passing each year at first attempt* not only increase in magnitude but also become significant for all the UKCAT scales under consideration.

| **Main predictor** | **Original data**  **Odds Ratios**  **(95% CIs)** | **MI data**  **Odds Ratios**  **(95% CIs)** |
| --- | --- | --- |
| *Verbal reasoning score* |  |  |
| Verbal reasoning | 1.04 (0.96, 1.13) | 1.07 (1.01, 1.17) |
| Advanced qualifications | 2.12 (1.83, 2.47) | 1.74 (1.56, 1.94) |
| Age | 0.97 (0.70, 1.36) | 1.05 (0.91, 1.22) |
| Advanced qualifications/Age interaction | 0.57 (0.45, 0.72) | 0.63 (0.52, 0.77) |
| Non-white ethnicity | 0.48 (0.41, 0.55) | 0.50 (0.45, 0.56) |
| Male sex | 0.66 (0.58, 0.75) | 0.73 (0.66, 0.80) |
| Time | 1.41 (1.35, 1.48) | 1.39 (1.34, 1.45) |
| Non-selective school attended | 1.14 (1.00, 1.31) | 1.12 (1.01, 1.24) |
| Non-professional social economic background | 1.10 (0.69, 1.77) | 0.88 (0.64, 1.21) |

Supplementary table S5. Results from a logistic regression predicting passing a year at medical school at first attempt (versus another academic outcome) implemented with a generalised estimating equations (GEE) framework. Results from multiply imputed datasets (m=30) are shown alongside those from the original data for comparison. In the case the main predictor is the *verbal reasoning* scale score of the UKCAT.

| **Main predictor** | **Original data**  **Odds Ratios**  **(95% CIs)** | **MI data**  **Odds Ratios**  **(95% CIs)** |
| --- | --- | --- |
| *Abstract reasoning score* |  |  |
| Abstract reasoning | 1.07 (1.00, 1.15) | 1.10 (1.04, 1.15) |
| Advanced qualifications | 2.04 (1.79, 2.33) | 1.72 (1.54 , 1.92) |
| Age | 0.98 (0.73, 1.32) | 1.06 (0.92, 1.22) |
| Advanced qualifications/Age interaction | 0.56 (0.45, 0.70 ) | 0.64 (0.52, 0.78) |
| Non-white ethnicity | 0.49 (0.43, 0.56) | 0.49 (0.44, 0.54) |
| Male sex | 0.67 (0.59, 0.76) | 0.74 (0.67, 0.81) |
| Time | 1.42 (1.36, 1.48) | 1.39 (1.34, 1.45) |
| Non-selective school attended | 1.20 (1.06, 1.35) | 1.12 (1.01, 1.24) |

Supplementary table S6. Results from a logistic regression predicting passing a year at medical school at first attempt (versus another academic outcome) implemented with a generalised estimating equations (GEE) framework. Results from multiply imputed datasets (m=30) are shown alongside those from the original data for comparison. In the case the main predictor is the *abstract reasoning* scale score of the UKCAT.

| **Main predictor** | **Original data**  **Odds Ratios**  **(95% CIs)** | **MI data**  **Odds Ratios**  **(95% CIs)** |
| --- | --- | --- |
| *Quantitative reasoning score* |  |  |
| Quantitative reasoning | 1.01 (0.92, 1.10) | 1.07 (1.01, 1.14) |
| Advanced qualifications | 2.13 (1.83, 2.48) | 1.73 (1.55, 1.93) |
| Age | 0.98 (0.70, 1.36) | 1.07 (0.65, 0.79) |
| Advanced qualifications/Age | 0.57 (0.45, 0.72) | 0.64 ( 0.52, 0.78) |
| Non-white ethnicity | 0.47 (0.41, 0.54) | 0.49 (0.44, 0.54) |
| Male sex | 0.66 (0.58, 0.76) | 0.72 (0.65, 0.79) |
| Time | 1.41 (1.35, 1.48) | 1.39 (1.34, 1.45) |
| Non-selective school attended | 1.14 (1.00, 1.31) | 1.12 (1.01, 1.24) |
| Non professional social economic background | 1.10 (0.69, 1.76 ) | 0.88 (0.64, 1.20) |

Supplementary table S7. Results from a logistic regression predicting passing a year at medical school at first attempt (versus another academic outcome) implemented with a generalised estimating equations (GEE) framework. Results from multiply imputed datasets (m=30) are shown alongside those from the original data for comparison. In the case the main predictor is the *quantitative reasoning* scale score of the UKCAT.

| **Main predictor** | **Original data**  **Odds Ratios**  **(95% CIs)** | **MI data**  **Odds Ratios**  **(95% CIs)** |
| --- | --- | --- |
| *Decision analysis score* |  |  |
| Decision analysis | 1.03 (0.95, 1.12) | 1.07 (1.02, 1.13) |
| Advanced qualifications | 2.12 (1.82, 2.47) | 1.73 (1.55, 1.93) |
| Age | 0.97 (0.70, 1.36) | 1.06 (0.92, 1.22) |
| Advanced qualifications/Age | 0.57 (0.45, 0.72) | 0.64 (0.52, 0.78) |
| Non-white ethnicity | 0.47 (0.41, 0.55) | 0.49 (0.45, 0.55) |
| Male sex | 0.66 (0.58, 0.76) | 0.73 (0.66, 0.80) |
| Time | 1.41 (1.35, 1.48) | 1.39 (1.34, 1.45) |
| Non-selective school attended | 1.15 (1.00, 1.31) | 1.12 (1.01, 1.25) |
| Non professional social economic background | 1.10 (0.68, 1.75) | 0.88 (0.64, 1.20) |
|  |  |  |

Supplementary table S8. Results from a logistic regression predicting passing a year at medical school at first attempt (versus another academic outcome) implemented with a generalised estimating equations (GEE) framework. Results from multiply imputed datasets (m=30) are shown alongside those from the original data for comparison. In the case the main predictor is the *decision analysis* scale score of the UKCAT.

| **Main predictor** | **Original data**  **Odds Ratios**  **(95% CIs)** | **MI data**  **Odds Ratios**  **(95% CIs)** |
| --- | --- | --- |
| *Total UKCAT score* |  |  |
| Total UKCAT score | 1.08 (0.99, 1.17) | 1.13 (1.07,1.21) |
| Advanced qualifications | 2.04 (1.78, 2.33) | 1.70 (1.52, 1.90) |
| Age | 0.98 (0.73, 1.31) | 1.07 (0.92, 1.23) |
| Advanced qualifications/Age | 0.55 (0.45, 0.70) | 0.64 (0.52, 0.78) |
| Non-white ethnicity | 0.50 (0.44, 0.57) | 0.50 (0.45, 0.56) |
| Male sex | 0.66 (0.58, 0.75) | 0.72 (0.66, 0.79) |
| Time | 1.42 (1.37, 1.48) | 1.39 (1.34, 1.45) |
| Non-selective school attended | 1.20 (1.06, 1.35) | 1.13 (1.07, 1.21) |
|  |  |  |

Supplementary table S9. Results from a logistic regression predicting passing a year at medical school at first attempt (versus another academic outcome) implemented with a generalised estimating equations (GEE) framework. Results from multiply imputed datasets (m=30) are shown alongside those from the original data for comparison. In the case the main predictor is the *total* scale score of the UKCAT.

.

**Adjusting for the effects for sociodemographic and educational variables**

The results relating to the ability of the UKCAT scores to predict *theory* performance, after adjusting for the effects of potential confounding educational and sociodemographic variables, are depicted in supplementary figure 3. As can be seen, the magnitude of the coefficients diminish after adjustment, but remain statistically significant in most cases. However, it can be observed that the scores for *abstract reasoning* do not independently and significantly predict performance on *theory* exams between years two and four. A trend for quantitative reasoning to increasingly independently predict theory performance over the course of undergraduate study can also be seen, though the absolute magnitude of the effect remains modest.

Similarly, the ability of the UKCAT scores to predict *skills*-based exams performance is reduced after adjustment for potential confounding variables (supplementary figure 4). In particular it can be observed that none of the components of the UKCAT are significantly predictive for skills performance in the third year of medical school.


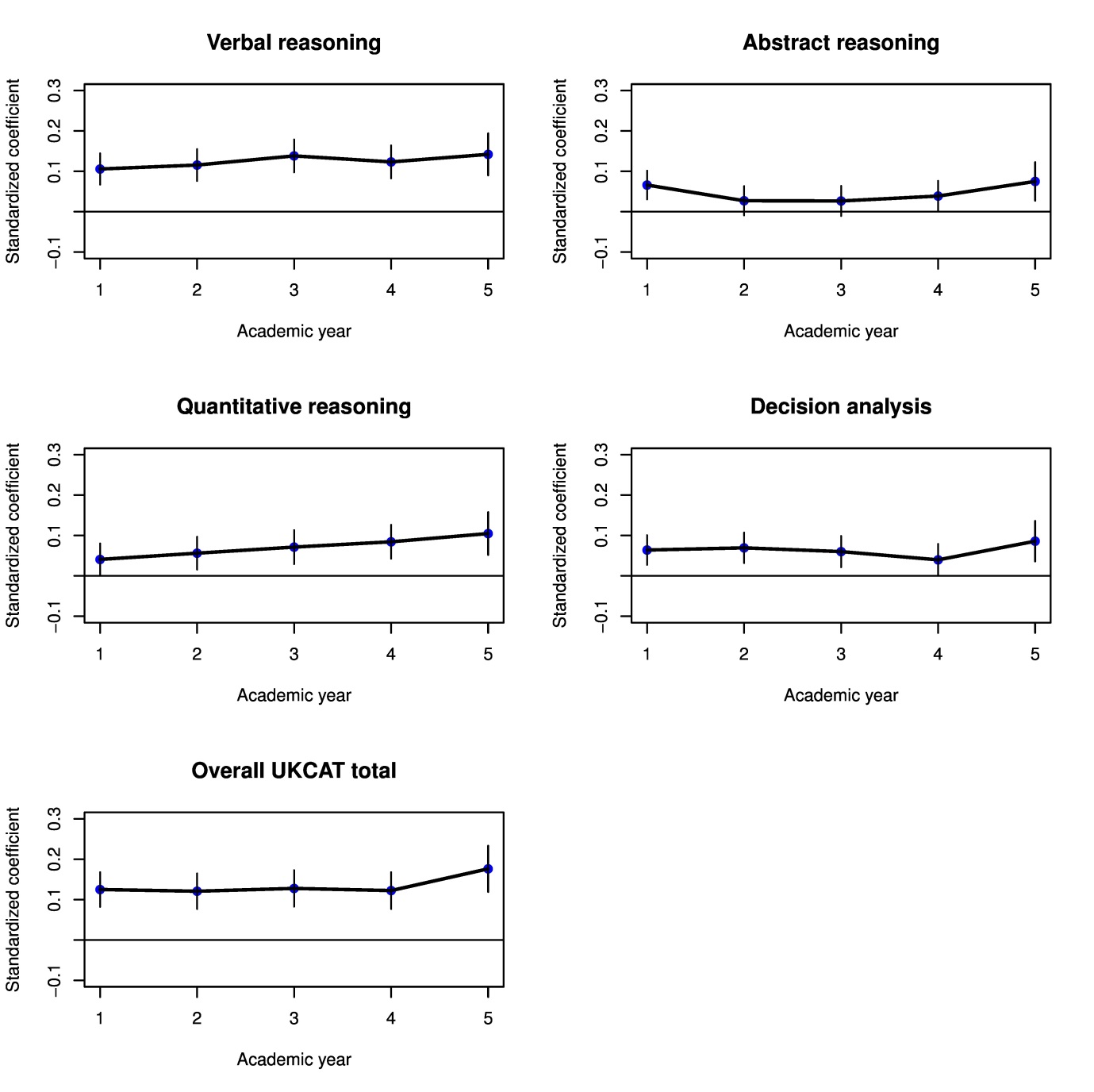


Supplementary figure 3. Results from a multilevel multivariable regression of performance on *theory* based medical school exams on the scales of the UKCAT, with standardised regression coefficients (and associated 95% confidence intervals) plotted.


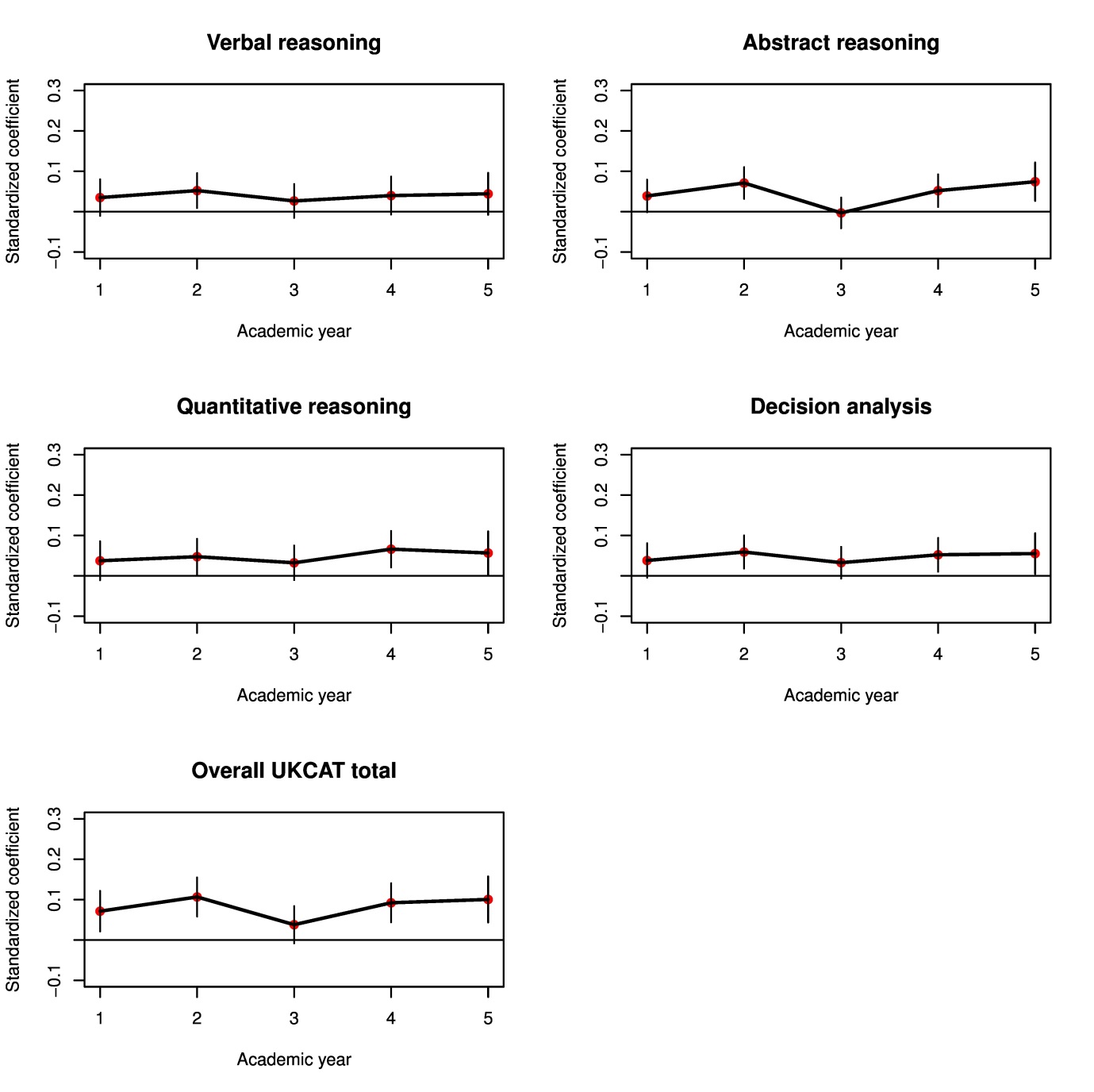


Supplementary figure 4. Results from a multilevel multivariable regression of performance on *skills* based medical school exams on the scales of the UKCAT, with standardised regression coefficients (and associated 95% confidence intervals) plotted.

| **UKCAT Scale** | **Odds Ratio ( 95% CI)** | **P value** |
| --- | --- | --- |
| Verbal reasoning | 1.04 (0.96, 1.13) | 0.31 |
| Quantitative reasoning | 1.01 (0.92, 1.10) | 0.89 |
| Decision analysis | 1.3 (0.95, 1.12) | 0.47 |
| Abstract reasoning | 1.07 (1.00, 1.15) | 0.04 |
| UKCAT total | 1.08 (0.99, 1.17) | 0.08 |

Supplementary table 5 Results of multivariable logistic regression (within a Generalised Estimating Equations framework) predicting the odds of passing each year at first attempt (compared to another academic outcome) according to standardised UKCAT score. The results are derived from 25638 undergraduate exam outcomes relating to 6812 medical students.

The independent odds of passing a year at medical school, as predicted by the UKCAT scores, are depicted in supplementary table 5. As can be seen, following adjustment for potential confounders, *abstract reasoning* score had the strongest independent ability to predict the odds of passing a medical school year at first attempt, though this was only of borderline statistical significance (OR 1.07, 95% confidence interval 1.00 to 1.15, p=0.04) there is around an 7% increased odds of passing first time for every standard deviation above the mean that is scored on the *abstract reasoning* scale of the UKCAT.

**References**

1 Emmanuel Lesaffre, and Andrew B Lawson, *Bayesian Biostatistics* (John Wiley & Sons, 2012).

2 Geert Molenberghs, and Michael Kenward, *Missing Data in Clinical Studies*. Vol. 61 (John Wiley & Sons, 2007).
